# Autophagy signal transduction by ATG proteins: from hierarchies to networks

**DOI:** 10.1007/s00018-015-2034-8

**Published:** 2015-09-21

**Authors:** Sebastian Wesselborg, Björn Stork

**Affiliations:** Institute of Molecular Medicine I, Heinrich-Heine-University, Universitätsstr. 1, Building 23.12, 40225 Düsseldorf, Germany

**Keywords:** Autophagy, ATG, ULK, PtdIns3K, LC3

## Abstract

Autophagy represents an intracellular degradation process which is involved in both cellular homeostasis and disease settings. In the last two decades, the molecular machinery governing this process has been characterized in detail. To date, several key factors regulating this intracellular degradation process have been identified. The so-called autophagy-related (ATG) genes and proteins are central to this process. However, several additional molecules contribute to the outcome of an autophagic response. Several review articles describing the molecular process of autophagy have been published in the recent past. In this review article we would like to add the most recent findings to this knowledge, and to give an overview of the network character of the autophagy signaling machinery.

**Electronic supplementary material:**

The online version of this article (doi:10.1007/s00018-015-2034-8) contains supplementary material, which is available to authorized users.

## Introduction

The term autophagy originates from the Greek expressions αùτóς (autos = self) and φαγεîν (phagein = to eat), literally meaning the self-eating of a cell. Next to the ubiquitin–proteasome system (UPS), autophagy is a major pathway for the degradation of intracellular cargo. Autophagy occurs at basal levels in any cell to carry out the proper degradation of long-lived proteins, protein aggregates or damaged organelles, ultimately ensuring cellular homeostasis. However, different stress conditions can cause the active induction of the autophagic machinery. These stress conditions include nutrient deprivation, growth factor withdrawal, hypoxia, or pathogen infection. Of note, the basic autophagic machinery is conserved among different eukaryotes, including yeast, animals, and plants.

## Types of autophagy and morphology

In 1962, Ashford and Porter observed cytoplasmic components, i.e., mitochondria or remnants thereof, in lysosomes of hepatic cells which had been perfused with glucagon [1]. In the same year, Novikoff and Essner observed similar mitochondria-containing vacuoles in hepatic cells from mice intravenously treated with the detergent Triton WR-1339 [2]. They termed these structures cytolysomes. In 1963, Christian de Duve suggested the name “autophagic vacuoles” for these cytolysomes and “autophagy” for the process of cellular self-eating [3].

Today, autophagy has become one of the most intensely investigated fields of cell biological research. This might partly be attributed to the fact that the process of autophagy or its dysregulation contribute to the onset of diverse human diseases or clinically relevant processes, including cancer, neurodegeneration, immune responses, or aging [4–7]. There exist three types of autophagy, i.e., macroautophagy, microautophagy, and chaperone-mediated autophagy [8]. Within chaperone-mediated autophagy, target proteins are directly recognized by cytosolic chaperones and transported across the lysosomal membrane [8]. Microautophagy describes a process by which the lysosomal membrane directly engulfs small portions of the cytoplasm [8]. During the process of macroautophagy (herein referred to as autophagy), cytoplasmic cargo is enveloped within a double-membraned vesicle, called autophagosome. Autophagosomes are transported to and fuse with lysosomes, leading to the generation of autolysosomes. Within autolysosomes, the sequestered cargo and the inner membrane of the autophagosome are degraded, and the resulting molecular building blocks such as amino acids or fatty acids are transported back to the cytosol through lysosomal permeases and are available for anabolic processes [9, 10]. Autophagy might be non-selective, leading to the bulk degradation of cytoplasm. However, in recent years different selective forms of autophagy have been identified and characterized, leading to the specific degradation of organelles or pathogens. These selective pathways include the autophagic degradation of mitochondria (mitophagy), peroxisomes (pexophagy), endoplasmic reticulum (reticulophagy or ER-phagy), ribosomes (ribophagy), protein aggregates (aggrephagy), lipid droplets (lipophagy), spermatozoon-inherited organelles following fertilization (allophagy), secretory granules within pancreatic cells (zymophagy), or intracellular pathogens (xenophagy) [11–14].

The formation of autophagosomes is a central hallmark of autophagy, and includes different discrete steps, i.e., nucleation, elongation and closure of the double-membraned vesicle. The cellular source of the autophagosomal membrane has been controversially discussed in the recent past. In yeast, a specific platform for the biogenesis of autophagosomes has been identified, the pre-autophagosomal structure (PAS) [15]. The PAS is a single punctate structure adjacent to the yeast vacuole, where most of the Atg proteins (see below) are present [8]. From the PAS the phagophore (also referred to as isolation membrane, IM) is generated, which envelopes cytoplasmic cargo to ultimately form the complete autophagosome [8]. In 2008, Axe et al. reported that the phosphatidylinositol 3-phosphate (PtdIns3P)-binding protein double FYVE domain-containing protein 1 (DFCP1) translocates to a punctate compartment upon nutrient starvation in mammalian cells [16]. The observed compartment is in dynamic equilibrium with the ER and provides a platform for the generation of the phagophore and the release of fully formed autophagosomes [16]. Since these structures were seen in association with the underlying ER forming an Ω-like shape, the authors termed them “omegasomes” [16]. Interestingly, so far no DFCP1 homolog has been reported for yeast [16]. Two further groups confirmed the physical connection between the ER and the phagophore by 3D electron microscopy [17, 18]. Collectively, these results strongly suggest that the phagophore originates from specialized subdomains of the ER. However, different other sources for autophagosomal membrane lipids have been suggested, including mitochondria, the Golgi complex, recycling endosomes, the nuclear envelope and the plasma membrane (reviewed in [8, 19, 20]). For example, it has been reported that mitochondria supply membranes for autophagosomes during starvation [21]. Apparently, autophagosome formation is dependent on ER/mitochondria connections. It has been proposed that these connections are necessary to transfer phosphatidylserine (PS) to the mitochondria, where PS is converted to phosphatidylethanolamine (PE). PE in turn is the target of Atg8/LC3-conjugation (described in “Two ubiquitin-like conjugation systems in autophagy: Atg12/ATG12–Atg5/ATG5 and Atg8/LC3–PE”). It has also been reported that autophagosomes themselves form at ER–mitochondria contact sites [22]. Recent data indicate that several specific organelles contribute to autophagosome formation, e.g., ER exit sites (ERES), coat protein II (COPII)-coated vesicles leaving the ERES, or the ER–Golgi intermediate compartment (ERGIC) [23–25]. Biazik et al. reported that forming phagophores can have multiple simultaneous membrane contact sites with surrounding organelles [26]. Presumably, different sources contribute to the completion of autophagosomes, presumably also depending on the autophagy-inducing stimulus and on the cargo to be degraded.

## Molecular regulation of autophagy

In the late 1990s, another era of autophagy research has evolved, leading to the molecular characterization of this process [27]. In 1993, Tsukada and Ohsumi reported the isolation and characterization of 15 *S. cerevisiae* mutants that displayed defective autophagy and named them *apg1*-*15* (autophagy) [28]. Similar screens were performed by other research groups, and the identified mutants defective in either autophagy, pexophagy, or the cytoplasm-to-vacuole pathway were called *aut*, *cvt*, *pdd*, *gsa*, *pag*, or *paz*, respectively [29–35]. In 2003, a unified nomenclature for the so-called autophagy-related genes/proteins, Atgs, was proposed [30]. Recently, yeast Atg39 and Atg40 have been identified as receptors which are apparently involved in the selective removal of the cytoplasmic and perinuclear ER and the nucleus [36]. Most of the yeast Atgs have homologs in the mammalian system (abbreviated as ATGs). However, sometimes homology is only based on function but not on sequence. Additionally, there exists one mammalian ATG, ATG101, which does not have an obvious counterpart in yeast [37, 38]. Frequently, in mammals different isoforms of a certain yeast Atg exist. Furthermore, different non-ATG proteins are involved in the regulation and process of autophagy, e.g., the mammalian/mechanistic target of rapamycin (mTOR), AMPK, AKT, AMBRA1, BCL2, DFCP1, or vacuolar protein sorting protein 34 (VPS34), which is the catalytic subunit of the class III phosphatidylinositol 3-kinase (PtdIns3K). Finally, different functions of ATGs in non-autophagic processes have been reported and are likely to emerge in the future (reviewed in [39]).

**Fig. 1:**
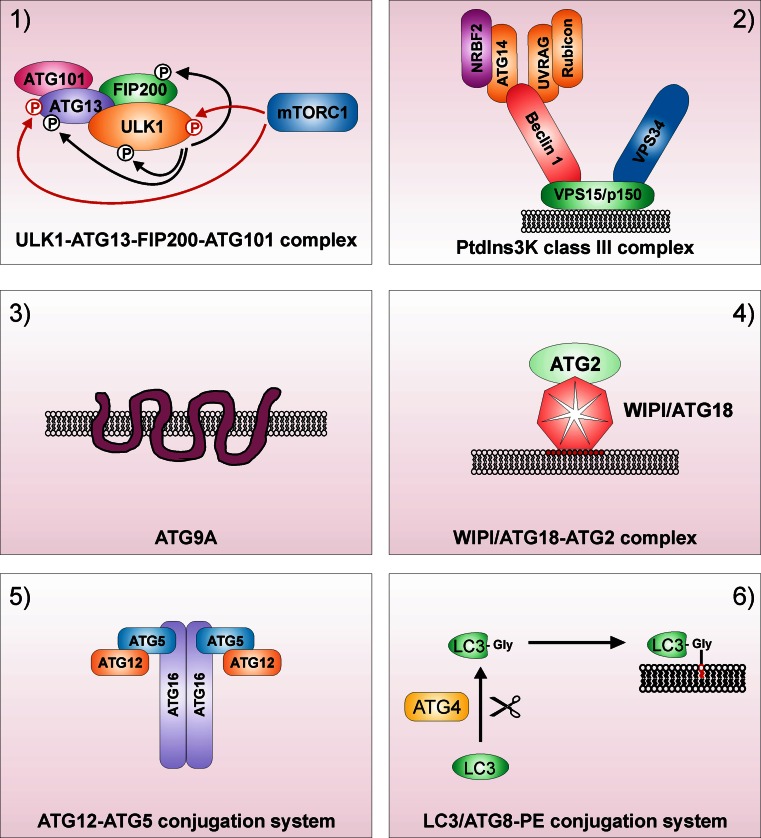
Functional clusters of autophagy signaling. **1** The ULK1–ATG13–FIP200–ATG101 protein kinase complex, **2** the PtdIns3K class III complex containing the core proteins VPS34, VPS15 and Beclin 1, **3** the multi-spanning transmembrane protein ATG9A, **4** the PtdIns3P-binding WIPI/ATG18–ATG2 complex, **5** the ubiquitin-like ATG5/ATG12 system and **6** the ubiquitin-like ATG8/LC3 conjugation system. For the ULK1 complex, mTOR-dependent inhibitory phosphorylations are depicted as *red arrows*, and ULK1-dependent activatory phosphorylations are depicted as *black arrows*. For the PtdIns3K class III complex, the mutually exclusive interactions of ATG14 or UVRAG with Beclin 1 are—for simplicity—shown within one complex

Functionally, mammalian ATGs can be subdivided in six functional clusters (Fig. 1): (1) the ULK1–ATG13–FIP200–ATG101 protein kinase complex; (2) the PtdIns3K class III complex containing the core proteins VPS34, VPS15 and Beclin 1; (3) the PtdIns3P-binding WIPI/ATG18–ATG2 complex; (4) the multi-spanning transmembrane protein ATG9A; (5) the ubiquitin-like ATG5/ATG12 system and (6) the ubiquitin-like ATG8/LC3 conjugation system (reviewed in [8]). These six modules regulate different steps during autophagosome biogenesis, i.e., vesicle nucleation, elongation of the autophagosomal membrane, and autophagosome completion. In the following, the autophagy-related functions of these six modules and their crosstalk will be described in detail, with the main focus laid onto the autophagy-initiating ULK1 kinase complex.

### The Atg1/ULK1 complex

#### The yeast Atg1–Atg13–Atg17 complex

In 1997, Matsuura et al. showed that the *apg1/atg1* gene discovered in their first screen for autophagy-defective yeast strains encodes a protein kinase (Apg1p/Atg1), and they reported an overall homology to *C. elegans* UNC-51 protein [40]. Furthermore, they showed that Atg1 overexpression suppressed the autophagy-defective phenotype in the *Δapg13/atg13* strain, indicating a linkage between Atg1 and Atg13 [41]. In the following years, the exact molecular details of the Atg1-dependent initiation of autophagy were deciphered. Yeast Atg1 associates with pathway-specific sets of Atg proteins, regulating either canonical autophagy or the yeast-specific cytoplasm-to-vacuole targeting (Cvt) pathway, respectively (reviewed in [42–45]). During canonical autophagy, Atg1 associates with Atg13, Atg17, Atg29 and Atg31. In contrast, during the Cvt pathway, Atg1 interacts with Atg11, Atg13, Atg20, Atg24 and Vac8 [44]. Accordingly, Atg17, Atg29 and Atg31 are selectively important for autophagy [46–48]. These three Atgs form a complex which is constitutively assembled and represents a scaffold for the recruitment of further Atgs to the PAS [49–51]. Upon starvation, Atg1 binds to Atg17, and this association is primarily mediated by Atg13 [52, 53]. Both the Atg1–Atg13 kinase complex and the autophagy-specific Atg17–Atg29–Atg31 complex cooperatively regulate the subsequent recruitment of downstream Atgs to the PAS, and for this function their physical interaction is mandatory [50, 54].

In 1998, it was reported that autophagy is negatively regulated by the protein kinase target of rapamycin (TOR), and that rapamycin accordingly induces the autophagic flux [55]. Two years later, Kamada et al. published a pioneering work demonstrating that this TOR-dependent control of autophagy is mediated by the Atg1 kinase complex [47]. The authors observed that both starvation and rapamycin enhanced the kinase activity of Atg1. Furthermore, Atg13 is hyperphosphorylated by TOR, resulting in a reduced affinity to Atg1. Accordingly, rapamycin treatment favors the dephosphorylation of Atg13 and its association with Atg1, resulting in increased Atg1 activity. Finally, the authors reported that rapamycin-induced Atg1 activity was decreased in the *Δatg17* strain, indicating that both Atg13 and Atg17 are important for Atg1 activation [47]. Subsequently the same group discovered that TOR phosphorylates Atg13 at S437, S438, S646, and S649. The authors mutated these four sites and four additional putative TOR sites (S348, S496, S535, S541) to alanines, and demonstrated that expression of the nonphosphorylatable Atg13-8SA mutant induced autophagy independently of TOR activity or nutrient status, apparently mimicking rapamycin treatment [56].

Notably, it has also been reported that Atg1 and Atg13 constitutively interact in vivo, irrespective of nutrient availability [57]. This situation would resemble the ULK1 complex constitution in higher eukaryotes (see below). Although the authors confirmed that binding of Atg13 to Atg1 indeed promotes its kinase activity and is important for efficient autophagy in vivo, the described observation would suggest that Atg1 activation in yeast is not exclusively controlled by regulated Atg13 binding, but rather involves additional levels of control. This could include conformational alterations or recruitment of additional factors regulated by the Atg13 phospho-status. Additionally, Atg1 phosphorylation itself is important for activation, as confirmed by two independent studies [58, 59]. However, next to TOR-regulation and Atg1 autophosphorylation additional kinases have been implicated in the regulation of the yeast Atg1–Atg13 complex, including PKA, Ksp1, Sch9 (yeast ortholog of mammalian AKT or p70S6K), or Snf1p [yeast ortholog of the mammalian AMP-activated protein kinase (AMPK)] [60–64]. Additionally, the phospho-status of the Atg1–Atg13 complex is likely to be regulated by phosphatases [65]. With regard to the downstream Atg1 substrates which regulate the initiation of autophagy in yeast, the current knowledge is less complete. Although different in vitro substrates have been identified for Atg1 by a global phosphorylation analysis, including Atg8 and Atg18, their in vivo relevance awaits further confirmation [66]. Previously it has been reported that Atg9 cycling depends on Atg1–Atg13 (described in “Atg9/ATG9A”), but apparently the kinase activity of Atg1 is not important [67]. Nevertheless, recently it has been reported that Atg1 can directly phosphorylate Atg9 and that this phosphosphorylation is required for the efficient recruitment of Atg8 and Atg18 to the site of autophagosome formation and subsequent expansion of the IM [68]. Identified Atg1 substrates are summarized in Table 1.

**Table 1:** Downstream targets of the Atg1/ULK1-complex

Target	P-site (species)	Function	References
**Yeast Atg1**			
Atg1	T226, S230 (*S. cerevisiae*; suggested as trans-autophosphorylation sites)	Essential for kinase activity	[58]
	T226 (*S. cerevisiae*)	Essential for kinase activity and autophagy	[59]
	11 Atg1-dependent sites (SILAC); S356, S390, S517 match consensus sequence (*S. cerevisiae*)	ND	[68]^a^
Atg2	4 Atg1-dependent sites (SILAC); S249, S1086 match consensus sequence (*S. cerevisiae*)	AA or DD mutant of S249, S1086: no effect on prApe1 processing	[68]^a^
Atg8	ND	ND	[66]
Atg9	7 Atg1-dependent sites (SILAC); S19, S802, S831, S948, S969 match consensus sequence; S657 matches consensus but was not detected by SILAC (*S. cerevisiae*)	Required for Cvt and autophagy (autophagosome formation); required for the efficient recruitment of Atg8 and Atg18	[68]^a^
Atg18	ND	ND	[66]
**Atg1/ULK1 in higher eucaryotes**			
AMBRA1	ND	Dissociation of PtdIns3K complex from dynein	[162]
	S465, S635 (*H. sapiens*)	ND	[124]
AMPK	α1: S360/T368, S397, S486/T488 β2: S38, T39, S68, S173 γ1: S260/T262, S269 (all *R. norvegicus*)	Reduced AMPK activation/activity	[143]
Atg1	ND (*D. melanogaster*)	Observation: increased by Atg13	[144]
Atg13	ND (*D. melanogaster*)	Observation: Atg1-dependent phosphorylation under fed and starvation conditions	[144]
	ND	ND	[107]
	ND	ND	[108]
	ND	ND	[109]
	ND	ND	[110]
	S48, T170, T331, T428, T478 (isoform 2, *H. sapiens*)	Not relevant for starvation-induced autophagy in chicken DT40 B cells	[122]
	S318 (isoform 2, *H. sapiens*)	Release from ULK1–Hsp90–Cdc37 complex; recruitment to damaged mitochondria	[123]
	S389 (isoform 1, *H. sapiens*)	ND	[124]
Atg101	S11, S203 (*H. sapiens*)	ND	[124]
Beclin 1	S14 (*M. musculus*; corresponds to human S15)	Increased activity of ATG14-containing VPS34 complex	[246]
	S15, S30, S96, S279, S337 (*H. sapiens*)	ND	[124]
DAPK3/ZIPK (Sqa)	T279 (in Sqa, *D. melanogaster*) ND for mammalian DAPK3/ZIPK3	Activation of myosin light chain kinase and thus activation of myosin II; regulation of Atg9 trafficking	[408]
FIP200	ND	ND	[108]
	ND	ND	[110]
	S943, S986, S1323 (*H. sapiens*)	ND	[124]
FUNDC1	S17 (*H. sapiens*)	Enhanced binding to LC3	[409]
p38α/MAPK	ND	Mediates transcription of IFN-stimulated genes	[167]
p62/SQSTM1	S405, S409 (*M. musculus*)	Increased binding affinity to ubiquitin	[361]
RAPTOR	ND	Inhibits the kinase activity of mTORC1	[154]
	Ser792, Ser855, Ser859, Ser863, Ser877	Inhibits the kinase activity of mTORC1	[153]
STING	S366 (*H. sapiens*)	Negative regulation of STING activity	[166]
Syntenin	S6	Prevents interaction of syntenin with ubiquitin	[410]
	S6, S61 (*H. sapiens*)	ND	[124]
ULK1	Between aa287 and aa351 (*M. musculus*)	ND	[78]
	T180 (*H. sapiens*)	Required for kinase activity	[128]
	S1047 (*M. musculus*)	Required for phosphorylation of S1043; promotes closed clamp conformation of ULK1	[119]
	ND	Help to maintain ULK1 in a closed conformation and to keep a dominant-negative motif at the C terminus inaccessible	[107]
	ND	Kinase-dead ULK1 acts as dominant-negative mutant	[82]
	ND	Observation: decreased in ATG13- or FIP200-depleted cells	[108]
	ND	Observation: increased in rapamycin-treated cells	[110]
VPS34	S249 (*H. sapiens*)	Not critical in VPS34 functional assays	[124]

*ND* not determined

^a^In this study, 32 Atg1 substrates were predicted, and 25 substrates were confirmed in vitro

In the last few years, the understanding of the signal transduction by the yeast Atg1 complex has significantly been complemented by several works investigating its structure. Ragusa et al. reported the crystal structure of a 2:2:2 complex of Atg17, Atg29 and Atg31 [69]. Atg17 is crescent-shaped with a 10 nm radius of curvature. During PAS organization and autophagy, the Atg17–Atg29–Atg31 complex dimerizes, and each dimer contains two complete crescents. The C-terminal “early autophagy targeting/tethering” (EAT) domain of Atg1 senses membrane curvature, dimerizes, and tethers lipid vesicles [69]. This double-crescent/S-shape architecture was also reported by Chew et al. [70]. The crystal structure of the N-terminal domain of yeast Atg13 has also been published [71]. Atg13 contains a HORMA (Hop1p, Rev7p, Mad2) domain at its N terminus. The HORMA domain is dispensable for the interaction with Atg1 or Atg13 recruitment to the PAS, but is apparently required for autophagy and the recruitment of the PtdIns3K subunit Atg14 (see “The PtdIns3K class III complex”) [71]. Furthermore, it has been reported that the Atg13 HORMA domain recruits Atg9 vesicles during autophagosome formation [72]. Fujioka et al. reported the X-ray crystallographic analysis of the interaction of yeast Atg13 with Atg1 and Atg17 [73]. Atg13 binds tandem microtubule interacting and transport (tMIT) domains in Atg1 via a 2-part MIT interacting motif (residues 460–521). Additionally, the Atg17-binding region was mapped to amino acids 424–436 of Atg13. The authors propose that starvation-induced dephosphorylation of specific serine residues in Atg13 enhances the interaction with both Atg1 and Atg17, directly explaining the TOR-dependent regulation of these interactions described above. The Atg1–Atg13 interaction was essentially confirmed by Stjepanovic et al. [74]. They report that Atg1–Atg13 complex binds as a unit to the Atg17–Atg31–Atg29 scaffold with ~10-μM affinity via Atg13. The resulting complex consists primarily of a dimer of pentamers in solution [74]. Recently, it has been shown that the PAS contains ~28 copies of Atg17 and—upon autophagy induction—similar numbers of Atg1 and Atg13 molecules [75]. Furthermore, they observe tetramers of Atg1 pentamers that assemble via Atg17–Atg31–Atg29, ultimately proposing a model for the higher organization of the Atg1 complex at the PAS [75]. Further aspects about the structural analyses of the Atg1 complex have been summarized in a recent review article by Noda and Fujioka [76].

#### The ULK1–ATG13–FIP200–ATG101 complex in higher eukaryotes

Atg1 has orthologs in the nematode *C. elegans* and the fruit fly *D. melanogaster*, i.e., UNC-51 and ATG1, respectively. In mammals, so far five orthologs have been identified, i.e., ULK1, ULK2, ULK3, ULK4, and STK36 (also termed Fused homolog) (reviewed in [42–45, 77]). In 1998, murine ULK1 was cloned and its similarity to yeast Atg1 and *C. elegans* UNC-51 was reported [78]. ULK1 consists of an N-terminal serine/threonine protein kinase domain, followed by a proline/serine (P/S)-rich domain and a conserved C-terminal domain (CTD). Shortly afterwards the same group reported the identification of human ULK1 and murine ULK2, respectively [79, 80]. In 2007, ULK1 was identified as an autophagy-modulating kinase by an siRNA screen of the kinome [81]. Knockdown of ULK1 in HEK293 cells blocked the autophagic response upon amino acid starvation or rapamycin treatment, respectively. ULK1 and ULK2 colocalize with ATG16L1 (see “Two ubiquitin-like conjugation systems in autophagy: Atg12/ATG12–Atg5/ATG5 and Atg8/LC3–PE”) and are accordingly targeted to the phagophore [82]. Notably, knockdown of ULK2 did not reveal any effect on autophagy induction in HEK293 cells, indicating that at least in this cellular system ULK1 and ULK2 cannot compensate each other [81]. However, compensatory roles of these two kinases can be deduced from the corresponding knockout mouse models. ULK1-deficient mice are viable and survive neonatal starvation periods [83]. Nonetheless, these mice reveal a delayed clearance of mitochondria from reticulocytes, indicating some differential roles of ULK1 and ULK2 for selective autophagy in general and for mitophagy in particular. Similarly, *Ulk2*^−/−^ mice are viable and do not show an overt autophagy phenotype [84]. In contrast, ULK1/2-double-deficient mice die shortly after birth, similar to mice deficient for ATG3, ATG5 or ATG7 [84]. Furthermore, autophagy induced by amino acid starvation is blocked in MEFs of these double-deficient mice [84]. The homology between ULK1 and ULK2 comprises the full length of the kinases, i.e., kinase domain, PS-rich domain, and CTD. In contrast, homology towards the other ULK family members is restricted to the kinase domain (reviewed in [42–45, 77]). However, ULK3 overexpression induced autophagy and premature senescence in a human fetal lung fibroblast cell line [85].

In 2007, Meijer et al. analyzed the degree of conservation for different Atgs/ATGs between different species [86]. They predicted that the protein KIAA0652 represents the human ortholog of yeast Atg13. Notably, they failed to identify Atg17 and Atg29 orthologs in higher eukaryotes [86]. Additionally, an Atg31 ortholog has not been reported in higher eukaryotes so far [8, 43]. However, in 2008 Hara et al. reported that the focal adhesion kinase family interacting protein of 200 kDa (FIP200) is an ULK1-interacting protein (Fig. 1, panel 1) [82]. Originally, FIP200 has been identified as a proline-rich tyrosine kinase 2 (Pyk2)- and focal adhesion kinase (FAK)-interacting protein which inhibits Pyk2 and FAK by direct binding to the kinase domains [87, 88]. FIP200 is also referred to as retinoblastoma 1-inducible coiled-coil 1 (RB1CC1) [89]. FIP200 is ubiquitously expressed and is involved in multiple cellular processes (reviewed in [90]). According to these multiple roles performed by FIP200, several FIP200-interacting proteins next to Pyk2 and FAK have been identified so far, including ATG16L1 (see “Two ubiquitin-like conjugation systems in autophagy: Atg12/ATG12–Atg5/ATG5 and Atg8/LC3–PE”), TSC1, p53, PP1, ASK1, TRAF2, Arkadia E3-ligase, COP1 E3-ligase, hSNF5, PIASy, β-catenin, ActA, and stathmin [91–105]. FIP200 comprises a putative nuclear localization signal (NLS) within the N-terminal half of the protein, a large coiled-coil domain and a leucine zipper motif at the C terminus [90]. FIP200 has been reported to localize in the cytoplasm, the nucleus and at focal adhesions [90]. The interaction between ULK1 and FIP200 requires the CTD of ULK1, and—similar to ULK1/2—FIP200 localizes to the phagophore upon starvation [82]. Furthermore, in FIP200-deficient MEFs autophagy induction is blocked, and the defect in autophagosome formation occurs downstream of mTOR [82]. Within the “Discussion” section of this first report demonstrating the importance of FIP200 for auto-phagy, the authors already speculate that FIP200 might represent the functional counterpart of yeast Atg17, due to several functional and architectural similarities [82, 106]. As described below, this assumption was subsequently confirmed by several reports.

Following the prediction by Meijer et al., several groups demonstrated that KIAA0652 indeed represents the human ATG13 ortholog (Fig. 1, panel 1) [107–110]. Furthermore, three of these reports deciphered the mechanistic details how mTOR regulates autophagy through the mammalian ULK1–ATG13–FIP200 complex. Human ATG13 is a 517 aa protein (isoform 1) and exhibits a 16 % identity to its yeast ortholog [109]. Chan et al. showed that knockdown of ATG13 blocks starvation-induced LC3 lipidation and ATG9A redistribution. They found that ATG13 binds to the CTD of Ulk1/2 [107]. Additionally, it was demonstrated that ATG13 serves as substrate for ULK1/2 and that the association between ULK proteins and ATG13 is not affected by the nutritional status or ATG13 phosphorylation [107]. In three almost simultaneously published studies, the mechanistic details how the mammalian ULK1–ATG13–FIP200 complex regulates autophagy and how mTOR transduces signals to this complex were elucidated [108–110]. ATG13 interacts with both ULK1/2 and FIP200 [108–110]. It appears that the association between ULK1 and FIP200 significantly depends on ATG13, but one group also demonstrated that ULK1 can independently interact with ATG13 and FIP200 [108]. Kim’s group reported that the last 75 aa of ATG13 are mandatory for ULK1/2 binding, and the last 134 aa for binding of both FIP200 and ULK1/2 [110]. We have recently fine-mapped the interaction sites between ATG13/ULK1 and ATG13/FIP200, respectively. It appears that the last three amino acids of ATG13 control binding to ULK1 and that the peptide sequence encoded by exon 14 of the human *ATG13* gene mediates binding to FIP200 ([111] and unpublished results). Size exclusion analyses by Mizushima’s group revealed that ULK1, ATG13 and FIP200 can be detected within a 3-MDa complex [109]. FIP200 is exclusively found in this mega-complex, and this complex cannot be detected in *FIP200*^−/−^ cells, indicating that FIP200 significantly contributes to the elution volume of this complex. The Jiang group performed size exclusion experiments with recombinant proteins and observed the three components within a complex with a molecular weight >1 MDa [108]. All three components of the complex localize to the phagophore upon induction of autophagy, and the assembly of the complex is not sensitive to starvation. Furthermore, ATG13 and FIP200 are required for maximal ULK1 kinase activity, ULK1 stability, and ULK1 recruitment to the phagophore. In turn, both ATG13 and FIP200 are substrates for ULK proteins. All three groups observed that either starvation or rapamycin treatment results in a faster migration of ULK1 and ATG13 in SDS-PAGE, and all three groups clearly demonstrated that mTOR phosphorylates both ULK1 and ATG13 [108–110]. Furthermore, Hosokawa et al. showed that the mTOR complex 1 (mTORC1) associates with the 3-MDa complex under nutrient-rich conditions and dissociates under starvation [109]. This interaction is mediated by the mTORC1 component RAPTOR and the PS-domain of ULK1. Of note, the mTORC1 binding site has alternatively been mapped to the kinase domain of ULK1 [112]. Accordingly, the ATG13-interacting CTD of ULK1 is not necessary for mTORC1 recruitment [109].

The fourth component of the ULK kinase complex has been identified and characterized independently by two groups. This component does not have any obvious ortholog in *S. cerevisiae* and was thus termed ATG101 [37, 38]. Of note, the closely related fission yeast *S. pombe* harbors an ATG101 ortholog (alternatively named Mug66) [37, 113–115]. ATG101 directly interacts with the ULK1 kinase complex through ATG13, and this association is independent of nutrient supply [37]. Mercer et al. mapped the ATG101-binding site in ATG13 to amino acids 112–220 [38]. In contrast to the results described above, the binding site of ATG13–ATG101 within ULK1 was mapped to the N-terminal half of the PS-rich domain, proximal to the kinase domain [38]. Notably, siRNA-mediated depletion of ATG101 suppresses GFP-LC3 puncta formation or GST-BHMT fragmentation, indicating that ATG101 is essential for autophagy [37, 38]. Although yeast and higher eukaryotes share some overlapping components of the Atg1/ULK1 complexes, e.g., Atg1/ULK1 itself and ATG13, there exist significant differences in complex constitution. As described above, yeast Atg11 and Atg17 serve as scaffolds during Cvt pathway or autophagy, respectively. It appears that FIP200 and ATG101 have overtaken some corresponding functions, since primary sequence orthologs of Atg11 and Atg17 do not exist in higher eukaryotes. Although FIP200 presumably represents a functional Atg17 ortholog [106], it should be noted that FIP200 is listed as Atg11 family member in the NCBI Pfam database and is structurally similar to *S. pombe* and *C. elegans* Atg11s [116]. Additionally, ATG101 has been reported to show similarity to yeast Atg17 [116].

To date, structural analyses of the ULK1 complex in higher eukaryotes remain less advanced compared to their yeast orthologs, but some interesting observations have recently been published. Suzuki et al. reported the structural analysis of the *S. pombe* Atg101–Atg13 complex [115]. The fission yeast *S. pombe* is a suitable model system for studying the mammalian ULK complex, since it conserves Atg1, Atg13, Atg17 and Atg101 orthologs, but not Atg29 and Atg31 [114, 115]. *S. pombe* Atg101 harbors a HORMA domain similar to that of Atg13. The HORMA-domain protein Mad2 has an open (O) and a closed (C) conformation, and it appears that Atg101 has a locked O-Mad2-like confirmation and stabilizes the C-Mad2-like conformation of Atg13. This in turn leads to the recruitment of downstream factors to the autophagosome formation site. In a parallel work, Michel et al. reported the crystal structure of human ATG101 [117]. The authors confirm the existence of ATG101 in a O-Mad2-like conformation. They also describe the presence of three large insertions relative to Mad2 (extensions 1, 2 and 3), which are all located to one pole of the molecule. Interestingly, extension 1 is missing in *S. pombe* Atg101, and extension 3 is significantly shorter [117]. The exact function of these extensions has to be unraveled in the future. Finally, the crystal structure of the ULK1 kinase domain in complex with different inhibitors has been reported by Lazarus et al. [118]. Whether the interaction mode between ATG13 and ULK1 is conserved in higher eukaryotes awaits further clarification, since (1) yeast and mammalian Atg13/ATG13 are not very homologous and (2) we observed that this interaction is—not necessarily directly mediated but at least—controlled by the last three amino acids of ATG13 [111].

Taking all the experimental observations summarized above into consideration, the following model has been established [Figs. 1 (panel 1), 2]: under nutrient-rich conditions, mTORC1 associates with the ULK1–ATG13–FIP200–ATG101 complex and phosphorylates ULK1 and ATG13. Under starvation conditions, mTORC1 dissociates from this mega-complex, and the inhibitory mTOR-dependent phospho-sites within ULK1 and ATG13 become dephosphorylated. Active ULK1 then autophosphorylates and phosphorylates ATG13 and FIP200, ultimately leading to the initiation of autophagosome formation. However, this proposed model leaves central remaining questions open, which will be partially addressed below or are currently being investigated: (1) how does mTOR-dependent phosphorylation of ULK1 and ATG13 keep the constitutively assembled complex in an inactive state; (2) which phosphatases dephosphorylate these inhibitory mTOR-sites and how does this contribute to the activation of the complex; (3) how is the phospho-status of ULK1 and ATG13 regulated in mTOR-independent pathways; (4) what is the role of the ULK-dependent phospho-sites in ATG13 and FIP200; (5) are additional interacting proteins and/or further post-translational modifications of this complex necessary for its autophagy-inducing function, and most importantly; (6) how does the ULK1–ATG13–FIP200–ATG101 complex initiate the downstream autophagy signaling machinery?

**Fig. 2:**
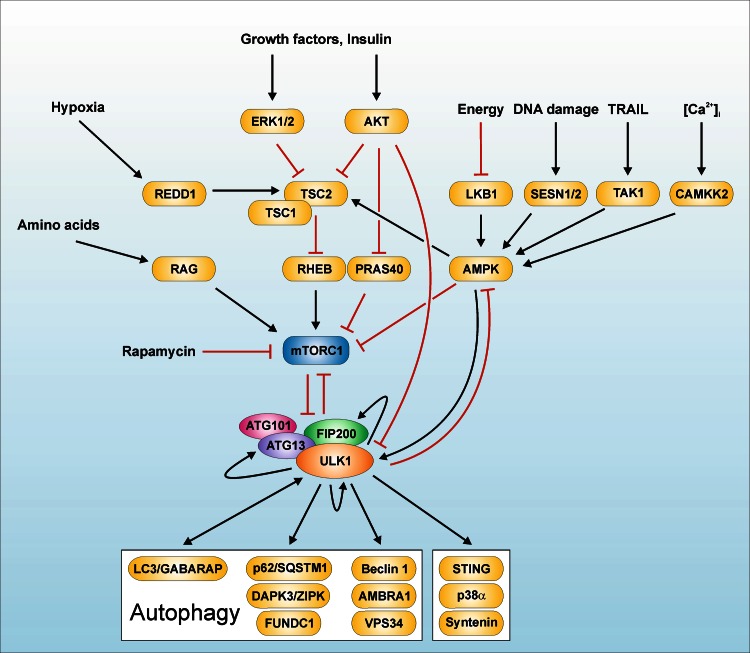
Signaling machinery upstream and downstream of the ULK1 complex. In recent years, the mTORC1-dependent regulation of the ULK1–ATG13–FIP200–ATG101 complex has been deciphered. Under nutrient-rich conditions, mTORC1 associates with the ULK1–ATG13–FIP200–ATG101 complex and phosphorylates ULK1 and ATG13. Under starvation conditions or upon treatment with mTOR inhibitors, mTORC1 dissociates from this mega-complex, and the inhibitory mTOR-dependent phospho-sites within ULK1 and ATG13 become dephosphorylated. Active ULK1 then autophosphorylates and phosphorylates ATG13 and FIP200, ultimately leading to the initiation of autophagosome formation [44, 45, 108–110]. The depicted substrates of ULK1 are listed in Table 1. MTOR has been established as central “gatekeeper” of autophagy, since this kinase integrates (1) nutrient signals, e.g., generated by growth factors or amino acids; (2) energy signals, e.g., controlled by the cellular AMP/ATP ratio; and (3) stress signals such as hypoxia or DNA damage. The Ser/Thr kinase mTOR is the catalytic subunit of two distinct kinase complexes, i.e., mTORC1 and mTORC2. The two complexes contain unique associated proteins which serve as scaffolds and determine the substrate specificity of the complexes, i.e., regulatory‐associated protein of mTOR (RAPTOR) and rapamycin‐insensitive companion of mTOR (RICTOR), respectively [396–398]. Next to these two proteins, the two complexes both harbor additional specific interacting proteins and share some components. Amino acids are sensed by the RAG family of small GTPases. Active RAG heterodimers translocate mTORC1 to lysosomal surfaces, where they bind to the so-called Ragulator complex [399]. On the surface of lysosomes, mTORC1 is activated by another small GTPase termed RAS-homologue enriched in brain (RHEB). The presence of growth factors is transmitted to mTOR via AKT. AKT phosphorylates tuberous sclerosis 2 protein (TSC2; also termed tuberin), which together with TSC1 (also termed hamartin) forms the TSC1–TSC2 complex. AKT-dependent phosphorylation of TSC2 inhibits the GTPase activating protein (GAP) activity of the TSC1–TSC2 complex for RHEB, thus promoting mTORC1 activation by GTP-loaded RHEB [400–402]. Alternatively, AKT phosphorylates PRAS40, which is subsequently bound by 14-3-3 proteins and cannot inhibit mTORC1 anymore [403–405]. Low energy levels as sensed by a high AMP/ATP ratio are transmitted to mTORC1 via AMPK. AMPK can—like AKT—phosphorylate TSC2. However, AMPK-dependent TSC2 phosphorylation leads to increased GAP activity of the TSC1-TSC2 complex and thus to mTORC1 inhibition [406, 407]. Alternatively, AMPK can directly inhibit mTORC1 by RAPTOR phosphorylation [137]. Stress signals like hypoxia, DNA damage, TRAIL or Ca^2+^ signals also inhibit mTORC1 via AMPK and/or the TSC1‐TSC2 complex (reviewed in [42, 126, 127]). Finally, AKT and AMPK can directly regulate ULK1, and ULK1 can—by negative feedback loops—regulate the upstream kinases mTORC1 and AMPK [120, 121, 128, 140, 141, 143, 153, 154]

#### Regulation of the ULK complex by post-translational modifications and downstream effectors

According to the above described model, the phospho-status of the ULK1–ATG13–FIP200–ATG101 complex is central for the regulation of autophagic processes. In general, global phosphorylation of ULK1 and ATG13 is decreased under starvation conditions and FIP200 phosphorylation is decreased under fed conditions [44, 45, 108–110]. In other words, it appears that the phospo-status of ULK1 and ATG13 primarily depends on mTOR, whereas the phospho-status of FIP200 mainly depends on ULK1, respectively. Several groups identified phosphorylation sites within ULK1 by mass spectrometry (see supplemental Table 1), both under nutrient-rich and starvation conditions [119–121]. These phospho-acceptor sites are distributed over the full length protein, i.e., within the kinase domain, the PS-rich domain and the CTD. These proteomic screens revealed that some of the sites are constitutively phosphorylated whereas others show a dependency on the nutritional conditions. With regard to ULK1-dependent sites in ATG13, seven phospho-acceptor sites have been published [121–123]. The SILAC-based approach by Shang et al. revealed that total phosphorylation levels of ATG13 were low under nutrient-rich conditions and stayed largely unaltered upon starvation [121]. The authors could only identify phosphorylation of ATG13 S361 (isoform 1), and phosphorylation of this site did not significantly change during starvation [121]. This would indicate that rather the ULK1 phospho-status than the ATG13 phospho-status governs autophagy initiation. Joo et al. showed that the Hsp90–Cdc37 chaperone complex regulates mitophagy by modulating ULK1 stability and function. They reported that ULK1-mediated phosphorylation of ATG13 at S318 (isoform 2; corresponds to S355 in isoform 1) is required for the release of ATG13 from an ULK1–Hsp90–Cdc37 complex and for the recruitment of ATG13 to damaged mitochondria, where it contributes to Parkin-mediated mitophagy (see “Two ubiquitin-like conjugation systems in autophagy: Atg12/ATG12–Atg5/ATG5 and Atg8/LC3–PE”) [123]. These results might account for the selective role of ULK1 for the mitochondrial clearance during reticulocyte development described above, and additionally the data suggest a phosphorylation-dependent regulation of the ULK1–ATG13 interaction during selective autophagy processes. We were able to identify five ULK1-dependent phospho-sites of ATG13 by an in vitro kinase assay (corresponding to S48, T170, T331, T428 and T478 in human isoform 2). However, mutation of these five sites did not alter starvation-induced autophagy in chicken DT40 B lymphocytes, although starvation-induced autophagy is completely blocked in *ATG13*^−/−^ DT40 cells [122]. Additionally, mTOR-dependent sites of mammalian ATG13 have not been reported so far [42, 45]. Recently, ULK1-dependent phospho-sites of FIP200 have been identified, but their functional relevance awaits further clarification [124].

On the basis of its central role for the regulation of the ULK1–ATG13–FIP200–ATG101 complex, mTOR has been dubbed the “gatekeeper” of autophagy (Fig. 2). The protein kinase mTOR integrates (1) nutrient signals, e.g., generated by growth factors or amino acids; (2) energy signals, e.g., controlled by the cellular AMP/ATP ratio; and (3) stress signals such as hypoxia or DNA damage (reviewed in [125–127]). The growth factor and energy inputs are essentially controlled by the serine/threonine protein kinases AKT and AMP-activated protein kinase (AMPK), which both function as upstream regulators of mTOR. Both kinases have also been implicated in the direct regulation of the ULK1 kinase complex (Fig. 2). The serine/threonine kinase AKT (also termed protein kinase B, PKB) translates signals received by receptor tyrosine kinases or receptor-associated tyrosine kinases into a diverse array of intracellular responses, including cell cycle control, metabolism, apoptosis, or autophagy. Bach et al. showed that ULK1 serves as direct substrate for AKT. The authors observed that insulin induces the AKT-dependent phosphorylation of ULK1 at S775 (human sequence) [128]. AMPK is the main sensor for cellular energy levels. AMPK consists of three subunits, i.e., the catalytic α-subunit and the regulatory β- and γ-subunits, respectively. Additionally, there exist several isoforms of the different subunits, i.e., α1–2, β1–2, and γ1–3 (reviewed in [129–131]). In order to exert its full catalytic activity, the α-subunit has to be phosphorylated within its activation loop at T172. Besides T172 phosphorylation of the α-subunit, the heterotrimeric AMPK complex is controlled by the regulatory β- and γ-subunits. In 2001, Wang and colleagues reported that the yeast AMPK-ortholog Snf1p is a positive regulator of autophagy and probably functions via Atg1 and/or Atg13, respectively [63]. Subsequently, several reports analyzed the role of AMPK for mammalian autophagy. Although an initial study reported that the AMPK-activating substances adenosine, AICA riboside and N6-mercaptopurine riboside inhibit autophagy [132], subsequently several works supported a positive regulatory role of AMPK for mammalian autophagy [133–136]. Generally, this positive effect of AMPK on autophagy has been attributed to its capability to inhibit mTOR (see Fig. 2). The inhibition of mTORC1 can be achieved by two different pathways, either by AMPK-mediated phosphorylation of the upstream regulator tuberous sclerosis complex 2 (TSC2) or by AMPK-mediated phosphorylation of the mTORC1-subunit RAPTOR (see Fig. 2) [137, 138]. However, in recent years a direct regulation of the ULK1–ATG13–FIP200–ATG101 complex by AMPK has been established, which is accordingly mTOR-independent. How does this direct regulation work? We and others demonstrated that AMPK directly interacts with ULK1 ([112, 120, 121, 139–142]. We discovered that ULK1 phosphorylates all three subunits of AMPK. It appears that ULK1-dependent phosphorylation of AMPK negatively regulates both its activation and activity, possibly establishing a negative regulatory feedback loop contributing to the termination of an autophagic response [143]. Interestingly, several groups reported that AMPK in turn phosphorylates ULK1 [120, 121, 140, 141]. However, different groups mapped different phospho-acceptor sites in the ULK1 amino acid sequence. Together with the proteomic screens analyzing the global nutrient-dependent ULK1 phosphorylation described above, a rather complex picture of the “ULK1 phospho-barcode” evolves. The different identified phospho-sites are summarized in supplemental Table 1 and reviewed in Wong et al. and Alers et al. [42, 45]. However, three ULK1 phospho-sites appear to be of particular interest, since they were reported by three or more independent groups, i.e., S556, S638, and S758 of human ULK1 sequence [45]. The identification of different sites by different groups already emphasizes that the AMPK-dependent regulation of the ULK1 complex is far from being completely characterized. However, it has been stated that the function of AMPK in autophagy is rather a “fine-tuning” than an “on–off switch” [45].

Apparently, mTOR, AKT, AMPK and presumably additional kinases (see prediction in [120]) contribute to the regulation of the ULK1–ATG13–FIP200–ATG101 complex. It is conceivable that these phosphorylation processes depend on different factors, i.e., cell type or autophagic stimulus. Additionally, there appear to exist significant differences between metazoan lineages in the regulation of the ULK kinase complex. Chang and Neufeld reported the regulation of this complex in *D. melanogaster* in parallel to the works on the mammalian complex. Interestingly, they reported common and divergent aspects. Like for the mammalian system, ATG13 is essential for starvation-induced autophagy, the ATG1–ATG13 interaction is independent of the nutrient status, and ATG13 is required for the autophagy-promoting function of ATG1 [144]. In contrast to the mammalian system, ATG13 is hyperphosphorylated under starvation conditions, indicating that the ATG13 phospho-status might be more dependent on ATG1 than on TOR in *D. melanogaster*. Furthermore, the authors demonstrated that ATG13 overexpression blocks autophagy and that TOR associates with ATG1/ATG13 independently of nutrient supply.

Next to the diversity among different species, different cell types and different autophagy-inducing stimuli, the complexity of this regulatory system is even increased by two additional aspects: (1) the action of phosphatases and (2) ULK1-dependent feedback signaling targeting the upstream kinases. It can be assumed that phosphatases contribute to the dephosphorylation of the mTOR-sites in ULK1 and ATG13, respectively [45]. Notably, the direct interaction between ULK1 complex components and protein phosphatases has been documented. For example, UNC-51 interacts with the protein phosphatase 2A (PP2A) in *C. elegans*, and FIP200 harbors a docking motif for protein phosphatase 1 (PP1) [100, 145]. However, the dephosphorylation of specific sites by specific phosphatases has not been reported yet. Generally, the phosphatase inhibitor okadaic acid is viewed as inhibitor of autophagy [45, 146–149]. Furthermore, PP2A has already been implicated in the regulation of autophagic processes, both as positive and negative regulator [65, 146, 148, 150]. However, it should be noted that PP2A enzymes fulfill multiple cellular functions with several different interacting proteins, and the involvement of additional or more selective phosphatases in the regulation of autophagy is currently being intensely investigated.

Next to phosphatase-mediated dephosphorylation processes, it has been postulated that feedback signaling pathways originating from the ULK kinase complex contribute to the shaping of an autophagic response. Apparently, ULK1 can directly influence its upstream regulators mTOR and AMPK, respectively (Fig. 2). As described above, we were able to identify ULK1-dependent regulation of AMPK. With regard to mTOR, it has been well documented that Atg1/ULK1 activity affects this kinase and its downstream signaling. In 2007, two groups independently reported that ATG1 overexpression in *D. melanogaster* negatively regulates the activity of the (m)TOR downstream target S6K [151, 152]. Similarly, Jung et al. observed an increased S6K phosphorylation upon knockdown of ATG13 or ULK1 [110]. Congruent to these observations, Chang and Neufeld reported that ATG1–ATG13 complexes regulate TOR by modulating its intracellular distribution and trafficking [144]. However, these reports only indirectly show the effect of Atg1/ULK1 on mTOR activity. Two reports proved that activated ULK1 directly phosphorylates RAPTOR and thus inhibits mTORC1 signaling [153, 154]. Collectively, these reports indicate that there is a close connection between mTOR-dependent cell growth control and autophagy signaling. Again another level of complexity is added by the fact that the ULK1 kinase complex component FIP200 interacts with TSC1, which is an upstream regulator of mTOR [92, 95]. Interaction of FIP200 with the TSC1–TSC2 complex results in the inhibition of this complex, ultimately leading to increased mTOR activity, S6K phosphorylation, and cell growth. Taken together, it appears that ULK1 and FIP200 have opposite effects on the regulation of mTOR activity, and future studies have to reveal the respective relative contributions.

In general, kinase-catalyzed phosphorylations and phosphatase-mediated dephosphorylations are the major molecular switches regulating the autophagy-initiating ULK1 complex. However, in the recent past alternative post-translational modifications have been implicated in this regulation, i.e., ubiquitination and acetylation. As described in “Two ubiquitin-like conjugation systems in autophagy: Atg12/ATG12–Atg5/ATG5 and Atg8/LC3–PE”, ubiquitination plays an essential role for cargo recognition during selective autophagy processes. Furthermore, this post-translational modification links the two major cellular degradation pathways, i.e., the ubiquitin–proteasome system (UPS) and selective autophagy. Meanwhile it is well established that interference with one pathway influences the flux through the other [155]. Finally, there are several lines of evidence that the ULK1 complex is modified by ubiquitin chains as well. Our group observed that treatment with the deubiquitinase inhibitor WP1130 increases ULK1 ubiquitination, and subsequently leads to the transfer of ULK1 to cellular aggresomes and to the parallel loss of ULK1 activity [156]. Zhou et al. reported that nerve growth factor (NGF) can induce the interaction of ULK1 with the NGF receptor TrkA [157]. This apparently occurs through K63-polyubiquitination of ULK1 and binding of ULK1 to p62, which then recruits ULK1 to TrKA receptor complexes. The study by Joo et al. described above reporting ULK1-catalyzed phosphorylation of ATG13 at S318 indirectly confirms ULK1 ubiquitination. The authors demonstrate that the disruption of the association between the Hsp90–Cdc37 chaperone complex and ULK1 by the Hsp90 antagonist 17-allyl-amino-17-demethoxygeldanamycin (17AAG) leads to ULK1 destabilization, which can be inhibited with the proteasome inhibitor MG132 [123]. Jiao et al. identified the chaperone-like protein p32 as a key regulator of ULK1 stability [158]. P32 forms a complex with ULK1, and p32 depletion increased K48-linked but decreased K63-linked polyubiquitination of ULK1, leading to proteasome-mediated degradation of ULK1. Li et al. reported that the mitochondrial outer-membrane E3 ligase MUL1 ubiquitinates ULK1 and regulates selenite-induced mitophagy [159]. Finally, Nazio et al. reported that mTOR does not only regulate the ULK complex by phosphorylation, but also indirectly by regulating ULK1 ubiquitination [160]. In this study the authors show that under basal conditions mTOR phosphorylates activating molecule in Beclin 1-regulated autophagy 1 (AMBRA1; alternatively named autophagy/Beclin 1 regulator 1) and thus keeps it inactive. AMBRA1 is a Beclin 1-interacting protein (see “The PtdIns3K class III complex”) [161]. Upon autophagy induction, AMBRA1 enhances ULK1 kinase activity and stability and promotes ULK1 self-association by enhancing K63 ubiquitination of ULK1 through the AMBRA1-associated E3-ligase tumor necrosis receptor-associated factor 6 (TRAF6) [160]. In turn, ULK1 phosphorylates AMBRA1 and thus promotes it detachment from the dynein complex [162]. Notably, Chang and Neufeld already observed that ATG1 and ATG13 levels were affected by TOR function in *D. melanogaster*, i.e., reduced levels in cells with high TOR activity and increased in cells with low TOR activity [144]. Generally, it appears that the components of the ULK1 complex are important for their mutual stabilization. ULK1 is destabilized in cells deficient for ATG13, FIP200 or ATG101 [37, 108–110]. Similarly, FIP200 is destabilized in cells deficient for ATG13 [109], and ATG13 is reduced in ATG101-depleted cells [37, 38].

Next to ubiquitination, acetylation has been reported to regulate autophagy. Gammoh et al. report that the histone deacetylase (HDAC) inhibitor suberoylanilide hydroxamic acid (SAHA) activates autophagy via the inhibition of mTOR and transcriptional up-regulation of LC3 expression [163]. The authors confirmed that the SAHA-mediated induction of autophagy depends on ULK1/2. Recently, the direct acetylation of ULK1 was reported. Lin et al. found that glycogen synthase kinase 3 (GSK3), which is activated by growth factor deprivation and resulting AKT inactivation, phosphorylates and thus activates acyltransferase TIP60 [164]. Activated TIP60 in turn acetylates and stimulates ULK1. ULK1 acetylation presumably occurs at K162 and/or K606, and a non-acetylatable ULK1 mutant failed to rescue autophagy in *Ulk1*^−/−^ MEFs [164]. Next to the direct AKT-mediated ULK1 phosphorylation described above, the GSK3–TIP60–ULK1 axis is another example how AKT-dependent signals are transduced to the ULK1 complex independently of mTOR.

With regard to the downstream signaling machinery, different ULK1 substrates have been reported (Fig. 2), but frequently their exact contribution to the induction of autophagy has still to be examined. Generally, the different ULK1 substrates can be grouped into different categories: (1) components of the ULK1 complex; (2) components of the PtdIns3K complex (see “The PtdIns3K class III complex”); (3) other autophagy-related regulators and proteins, and (4) non-autophagy-related substrates (Table 1; Fig. 2). There likely exist several additional ULK1 substrates which contribute to the regulation of the autophagic flux in a phosphorylation-dependent manner. The identification and characterization of these ULK1 substrates will greatly enhance our understanding of autophagy signaling pathways. Furthermore, it has to be noted that there apparently exist kinase-independent autophagic ULK1 functions, and non-autophagic functions of ULK1 complex components (reviewed in [45]) (Fig. 2). Several observations have recently been reported to underscore the latter aspect: nuclear ULK1 can promote cell death in response to oxidative stress [165], ULK1 can negatively regulate the stimulator of interferon genes (STING) pathway [166], and ULK1 mediates expression of interferon-stimulated genes via the p38alpha MAPK pathway [167]. Finally, also ULK1/2-independent autophagic processes have been reported [84, 122]. The signaling machinery upstream and downstream of the ULK1 complex is summarized in Fig. 2.

### The PtdIns3K class III complex

#### The yeast PtdIns3K class III complexes

Next to the ULK1–ATG13–FIP200–ATG101 complex, another multiprotein-complex is important for the formation of autophagosomes. In yeast, the class III phosphatidylinositol 3-kinase (PtdIns3K class III) Vps34 functions in both autophagy and sorting of vacuolar proteins. Two separate Vps34 subcomplexes have been identified to mediate these functions [168]. The autophagy-regulating complex I contains Vps34, Vps15, Atg6 (Vps30), and Atg14. In contrast, the sorting of vacuolar proteins is mediated by complex II, which contains Vps38 instead of Atg14 [168]. Accordingly, the unique complex-subunits Atg14 and Vps38 regulate the intracellular localization and the specific functions of these two complexes. Atg14 mediates the localization of complex I to the PAS, whereas Vps38 controls the localization of complex II to endosomes [169, 170]. Recently, Araki et al. reported the identification and characterization of yeast Atg38 [171]. The authors describe that Atg38 physically interacts with Atg14 and Vps34 via its N terminus. The C terminus of Atg38 mediates homodimerization, which is indispensable for the integrity of complex I. Accordingly, it appears that the homodimer of Atg38 functions as a linker between the Vps15–Vps34 and Atg14–Atg6 subcomplexes, ultimately facilitating complex I formation [171].

#### Mammalian PtdIns3K class III complexes

Similar to the situation in yeast, different PtdIns3K class III complexes could be identified in mammals (reviewed in [172–176]). The mammalian PtdIns3K class III core complex consists of the catalytic subunit VPS34, the adaptor VPS15 (p150), and Beclin 1 (ATG6) [Figs. 1 (panel 2), 3]. Beclin 1 forms the scaffold for the recruitment of additional activators or repressors of the PtdIns3K class III complex. Beclin 1 contains an N-terminal intrinsically disordered region, a BCL2 homology 3 (BH3) domain, a coiled-coil domain, and a C-terminal β-α repeated, autophagy-specific (BARA) domain [175]. Additionally, He et al. identified another mammalian ortholog of Beclin 1—termed Beclin 2—which functions in both autophagy and the degradation of G protein-coupled receptors [177]. Although we address some of the regulatory signaling pathways targeting and established by Beclin 1, we refer readers to other excellent reviews on this molecule [172–175].

**Fig. 3:**
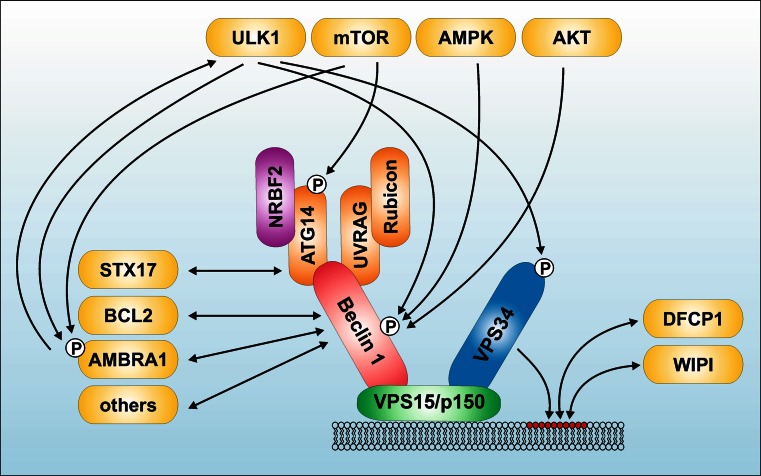
Signaling machinery upstream and downstream of the PtdIns3K class III complex. The PtdIns3K class III core complex consists of the catalytic subunit VPS34, the adaptor VPS15 (p150), and Beclin 1 (ATG6). Beclin 1 binds to additional regulatory proteins, including ATG14, NRBF2, UBRAG, Rubicon, AMBRA1, BCL2, and several others (reviewed in [172–175]). Furthermore, there exists considerable crosstalk between the ULK1-complex and the PtdIns3K class III complex (for details see “The PtdIns3K class III complex”). ULK1 phosphorylates Beclin 1, AMBRA1, and VPS34. In turn, AMBRA1 regulates ULK1 stability and activation. Next to ULK1 itself, several ULK1-regulating kinases—such as mTORC1, AMPK, and AKT—also regulate the PtdIns3K class III complex. The product of the PtdIns3K class III catalytic activity is phosphatidylinositol 3-phosphate (PtdIns3P) (*red circles*). PtdIns3P then recruits the downstream effectors DFCP1 and proteins of the WIPI-family. For simplicity, the mutually exclusive interactions of ATG14 or UVRAG with Beclin 1 are shown within one complex

In the recent past, three major subcomplexes have been reported, which contain either ATG14, UV radiation resistance-associated gene protein (UVRAG), or a dimer of UVRAG and RUN domain protein as Beclin 1 interacting and cysteine-rich containing (Rubicon) [Figs. 1 (panel 2), 3].

ATG14 (alternatively called Atg14-like, ATG14L, or Beclin 1-associated autophagy-related key regulator, Barkor) is the putative mammalian homolog of yeast Atg14 and was identified by four different groups [178–181]. Accordingly, the ATG14 containing PtdIns3K class III complex likely represents the functional equivalent to yeast complex I. ATG14 co-localizes with several marker proteins on phagophores, indicating that this complex is involved in an early stage of autophagy. Furthermore, ATG14-silencing suppresses autophagosome formation [178–181]. It has additionally been shown that ATG14 increases VPS34 catalytic activity in a Beclin 1-dependent manner [181]. Binding of ATG14 to Beclin 1 is mediated via their respective coiled-coil domains [179–181]. Most interestingly, recently it was shown that ATG14 is not only involved in early steps of autophagosome formation, but also in later steps. Diao et al. reported that ATG14 promotes membrane tethering and fusion of autophagosomes to endolysosomes [182]. This ATG14 function requires ATG14 homo-oligomerization by its cysteine repeats. In contrast, this homo-oligomerization is not required for initial autophagosome formation [182]. Apparently, ATG14 binds to the soluble *N*-ethylmaleimide-sensitive factor attachment protein receptor (SNARE) core domain of syntaxin 17 (STX17) and stabilizes the STX17–SNAP29 binary target-SNARE complex on autophagosomes (Fig. 3) [182]. Previously, Itakura et al. demonstrated that the SNARE protein Stx17 translocates to the outer autophagosomal membrane [183]. Fusion with lysosomes is then mediated by the interaction between autophagosome-resident Stx17, synaptosomal-associated protein 29 (SNAP-29), and the lysosome-resident vesicle-associated membrane protein 8 (VAMP8) [183].

In parallel, all four groups reported that the interactions of ATG14 or UVRAG with the PtdIns3K class III core complex are mutually exclusive, which is probably due to their overlapping binding sites in Beclin 1 [178–181], and accordingly it has been suggested that UVRAG represents the mammalian Vps38 [178, 184]. Along these lines, UVRAG was shown to primarily associate with Rab9-positive late endosomes and partially with Rab5/Rab7-positive endocytic compartments, and UVRAG knockdown did not influence autophagic flux and GFP-LC3 dot formation (see “Two ubiquitin-like conjugation systems in autophagy: Atg12/ATG12–Atg5/ATG5 and Atg8/LC3–PE”) [178]. In contrast, UVRAG has originally been attributed a role in autophagy signaling [185]. Furthermore, Takahashi et al. demonstrated that Bif-1 (also termed endophilin B1) interacts with Beclin 1 through UVRAG, and that loss of Bif-1 suppresses autophagosome formation [186]. In parallel, Liang et al. suggest that UVRAG-mediated activation of the Beclin 1/VPS34 complex suppresses the proliferation and tumorigenicity of human colon cancer cells, and Takahashi et al. observed that Bif-1 knockout enhances spontaneous tumor development [185, 186]. However, the interplay between UVRAG-dependent autophagy and tumor suppression has also been controversially discussed. Knævelsrud et al. demonstrated that UVRAG mutations associated with microsatellite unstable colon cancer do not affect autophagy [187]. Taken together, the role of UVRAG for initial stages of autophagy remains rather elusive. In 2008, Liang et al. reported that UVRAG interacts with the class C Vps complex, which is a key component of the endosomal fusion machinery [188]. This interaction promotes the GTPase activity of Rab7 and autophagosome fusion with late endosomes/lysosomes. The authors also showed that UVRAG enhanced endocytic trafficking, directly supporting the above described UVRAG localization studies. Most interestingly, the effect on autophagosome maturation was independent of Beclin 1, indicating that UVRAG might play a dual role in autophagy regulation: (1) in combination with Beclin 1 during autophagosome formation and (2) in combination with C Vps/Rab7 during autophagosome maturation [188].

Finally, two of the four groups additionally identified Rubicon as negative regulator of autophagy [179, 181] [Figs. 1 (panel 2), 3]. Rubicon is alternatively called Beclin 1 associated RUN domain-containing protein (Baron) [189]. Rubicon was only found in UVRAG-containing Beclin 1/VPS34-complexes, but not in ATG14-containing ones. Furthermore, Rubicon knockdown also affected rather autophagosome maturation and endocytic trafficking [179, 181]. However, Zhong et al. observed that Rubicon inhibits VPS34 kinase activity only in the absence of Beclin 1 overexpression, suggesting that the negative regulatory role exerted by Rubicon is Beclin 1-independent [181]. Supporting this notion, it has been speculated that Rubicon interferes with pro-autophagic Rab GTPases via its RUN domain, and that sequestering of Rubicon by Beclin 1 would vice versa promote autophagy [172]. Currently, the dynamics of the above described complexes are intensively being investigated.

Recently, the putative mammalian counterpart of yeast Atg38 has been identified and independently reported by three different groups, which is named nuclear receptor binding factor 2 (NRBF2) [Figs. 1 (panel 2), 3]. Two groups reported that NRBF2 positively regulates autophagy, whereas one group observed autophagy-suppressing effects [190–192]. Accordingly, the autophagy-regulating capability of NRBF2 is not entirely clarified. Of note, NRBF2 was originally identified as interaction partner of nuclear receptors [193, 194]. Similar to its effect on autophagy, both activating and repressing effects of NRBF2 on nuclear receptor signaling have been reported [193, 194].

Baskaran et al. reported the structure of the ATG14-containing PtdIns3K complex as determined by single-particle electron microscopy [195]. It appears that the complex is V-shaped, with VPS15 at the base of the V and serving as bridge for VPS34 and the ATG14/Beclin 1 subcomplex.

#### Beclin 1-interacting proteins

Next to the stable binding of ATG14, UVRAG and Rubicon to Beclin 1, multiple cellular and viral Beclin 1-interacting proteins have been identified which bind rather transiently or specifically under certain conditions (reviewed in [172–175]) (Fig. 3). In the following, we would like to focus on the association of Beclin 1 with AMBRA1 and viral and cellular BCL2 homologs. However, additional Beclin 1-interacting proteins include EGFR [196], estrogen-receptor [197], FYVE-CENT [198], HMGB1 [199, 200], MyD88/TRIF [201], nPIST [202], PINK1 [203], Rab5 [204], SLAM [205], survivin [206], and VMP1 [207], or the viral proteins HIV NEF [208], HSV-1 ICP34.5 [209], and the influenza virus M2 protein [210].

AMBRA1 is a scaffolding protein with a molecular mass of ~130 kDa [161]. Next to the above described regulation of ULK1, AMBRA1 itself is regulated by ULK1-dependent phosphorylation (see below) (Fig. 3). A recent study by Fimia and colleagues showed that the interaction between AMBRA1 and Cullin E3 ubiquitin ligases regulates the dynamics of autophagic responses [211]. Under fed conditions, Cullin-4 binds to AMBRA1 and reduces its abundance. Under pro-autophagic conditions, ULK1 phosphorylates AMBRA1, leading to its dissociation from Culin-4. Stabilized AMBRA1 in turn can bind to Cullin-5, which leads to the accumulation of the mTOR-inhibitory protein DEPTOR. Under prolonged autophagic conditions, Cullin-4 reassociates with AMBRA1, leading to its degradation and the termination of the autophagic response [211]. However, AMBRA1 is not only involved in bulk autophagy processes, but also in mitophagy, cell death, cell proliferation, and development (reviewed in [212–214]). Of note, Cianfanelli et al. recently reported that AMBRA1 regulates the dephosphorylation and degradation of the proto-oncogene c-Myc via PP2A [215].

The association between Beclin 1 and viral and cellular BCL2 homologs establishes a direct connection between apoptosis and autophagy signaling pathways [216–224] (Fig. 3). Beclin 1 was originally identified as BCL2-interacting protein by a yeast-two-hybrid screen [219]. The functional relevance of this interaction has been described by Pattingre et al. in 2005 [222]. They showed that BCL2 can inhibit starvation-induced and Beclin 1-dependent autophagy. This has been confirmed for viral BCL2 proteins [216, 218, 222, 223]. The BH3 domain of Beclin 1 binds to the hydrophobic BH3-binding cleft of BCL2 [217, 220, 221, 225]. Although the interaction between Beclin 1 and BCL2 inhibits autophagy induction by nutrient deprivation, Beclin 1 does not suppress the anti-apoptotic function of BCL2, as would be expected from “classical” BH3-only proteins [226].

The interaction between Beclin 1 and BCL2 is regulated by several stimuli, including competitive binding, self-association, phosphorylation, or ubiquitination [174]. The Beclin 1 BH3 domain might be competitively displaced by other BH3-only proteins or by BH3 mimetics, e.g., ABT737 [220, 225, 227]. Alternatively, membrane-anchored receptors or adaptors, e.g., IP_3_Rs or toll-like receptor-associated Myd88/TRIF, might induce the disruption of the Beclin 1-BCL2 interaction [173]. Finally, it has also been reported that reactive oxygen species promote cytosolic translocation of high mobility group box 1 (HMGB1), where it interacts with Beclin 1 and thus displaces BCL2 [200]. It has also been discussed that Beclin 1-homo-oligomerization might provide a scaffold for further protein–protein interactions and displacement of BCL2 proteins [174]. Additionally, post-translational modifications of both interacting proteins might modulate the Beclin 1-BCL2 interaction. Interestingly, both components serve as phospho-acceptor proteins. Zalckvar et al. reported that the death-associated protein kinase (DAPK) phosphorylates Beclin 1 at T119, which is located within the BH3 domain [228]. In turn, BCL2 might be phosphorylated by the mitogen-activated protein kinases ERK and JNK, respectively. Wei et al. reported that JNK phosphorylates T69, S70 and S87 within the non-structured loop between BH3 and BH4 of BCL2 [224]. Next to the direct displacement of BCL2 by HMGB1 described above, it has been suggested that HMGB1 promotes the activation of ERK1/2, resulting in the ERK1/2-mediated phosphorylation of BCL2 and its dissociation from Beclin 1 [200]. Interestingly, it has been reported that viral BCL2 proteins inhibit autophagy more effectively than cellular BCL2 proteins. This has been explained by either a stronger affinity of viral BCL2 proteins to Beclin 1 or the fact that viral BCL2 orthologs lack the JNK-dependent phosphorylation sites described above [218, 223, 224, 229]. It appears that the BCL2-dependent blockade of autophagy might be a viral strategy to ensure latency. Finally, it was demonstrated that K117 within the BH3 domain of Beclin 1 is a major ubiquitination site [230]. Accordingly, the authors speculate that TRAF6-mediated K63-linked ubiquitination at this site influences the association between Beclin 1 and BCL2.

The interaction between BCL2 and Beclin 1 occurs both at the mitochondrion and at the ER, and both mitochondrion- and ER-targeted BCL2 reduce LC3-II accumulation induced by overexpression of Beclin 1 [231]. However, starvation-induced autophagy is most efficiently inhibited by ER-localized BCL2 [222, 231]. In 2009, Vicencio et al. reported the identification of a trimeric complex consisting of IP_3_Rs, Beclin 1 and BCL2 [232]. Apparently IP_3_Rs facilitate the interaction between Beclin 1 and BCL2, thus indirectly impairing autophagy. Upon IP_3_R inhibition, this trimeric complex dissociates and autophagy is induced. The authors further suggest that the Ca^2+^ channel function of the IP_3_Rs is not contributing to the autophagy-inhibitory effect [232]. However, this has been challenged by other groups [233, 234]. For example, in DT40 cells deficient for all three IP_3_Rs, association between Beclin 1 and BCL2 is not affected [235]. Notably, Khan et al. state that the absence of IP_3_Rs in the triple-knockout DT40 cells results in higher levels of basal autophagy, which would confirm the results by Vicenco et al. However, reconstitution with a functionally inactive D2550A IP_3_R mutant did not result in a suppression of the autophagic flux, indicating that the Ca^2+^ channel function of IP_3_Rs is important for the regulation of autophagy [235]. Along these lines, Decuypere et al. suggest that IP_3_R-mediated Ca^2+^ signaling and autophagy induction are indeed two interrelated processes [236]. They showed that IP_3_Rs are sensitized upon starvation, and that this sensitization depends on Beclin 1. In their model, Beclin 1 shuttles from BCL2 to the ligand binding domain of the IP_3_Rs upon starvation, indirectly confirming the importance of ER-localized BCL2 to modulate autophagy (see above). Next to IP_3_Rs, another ER-localized transmembrane protein has been implicated in the regulation of the Beclin 1-BCL2 association. Chang et al. reported the identification of the nutrient-deprivation autophagy factor-1 (NAF-1), and its requirement for BCL2 at the ER to functionally antagonize Beclin 1-dependent autophagy [237]. Additionally, NAF-1 also interacts with IP_3_Rs. Interaction with IP_3_Rs was also shown for different BCL2 family members (reviewed in [233, 234, 238–240]. Future studies will have to further elucidate the interplay between IP_3_Rs, other ER-localized proteins, BCL2 family members, and Beclin 1. However, a central role for the regulation of autophagy has also been attributed to mitochondria-localized BCL2. Strappazzon et al. showed that the positive autophagy regulator AMBRA1 preferentially binds to the mitochondrial pool of BCL2. Upon starvation, AMBRA1 is released and competes with BCL2 for binding to mitochondria- or ER-localized Beclin 1 [241]. Taken together, it appears that BCL2 proteins interfere with Beclin 1 function by at least two different ways, i.e., directly by binding of Beclin 1 or indirectly by binding to the positive regulator AMBRA1 [241, 242].

To date, different models have been brought up to explain the direct BCL2-dependent inhibition of Beclin 1 [243]. Pattingre et al. detected that BCL2 overexpression interferes with the formation of the Beclin 1–VPS34 complex [222]. Furthermore, they confirmed that the functional activity of the PtdIns3K class III complex is reduced. Recently, Wei et al. reported that mitogen-activated protein kinase-activated protein kinase 2 (MAPKAPK2) and MAPKAPK3 positively regulate starvation-induced autophagy by phosphorylating Beclin 1 at serine 90 [244]. The authors suggest that BCL2 can block this phosphorylation and thus inhibits autophagy. Noble et al. demonstrated that Beclin 1 forms a dimer in solution, which is bound by BCL2 proteins. UVRAG disrupts this Beclin 1 dimer interface and thus UVRAG–Beclin 1 heterodimers are assembled, which presumably cause the activation of autophagy. In turn, BCL2 proteins reduce the affinity of UVRAG for Beclin 1 and thus stabilize Beclin 1 homodimers [245]. Generally, only ATG14, UVRAG and Rubicon are stably associated with the PtdIns3K class III core complex [172]. Accordingly, the unstable or transient interaction of Beclin 1 with Bcl-2 proteins allows the dynamic regulation of autophagic processes.

#### Crosstalk between ULK1 and PtdIns3K class III complexes and downstream effectors of PtdIns3P

**Fig. 4:**
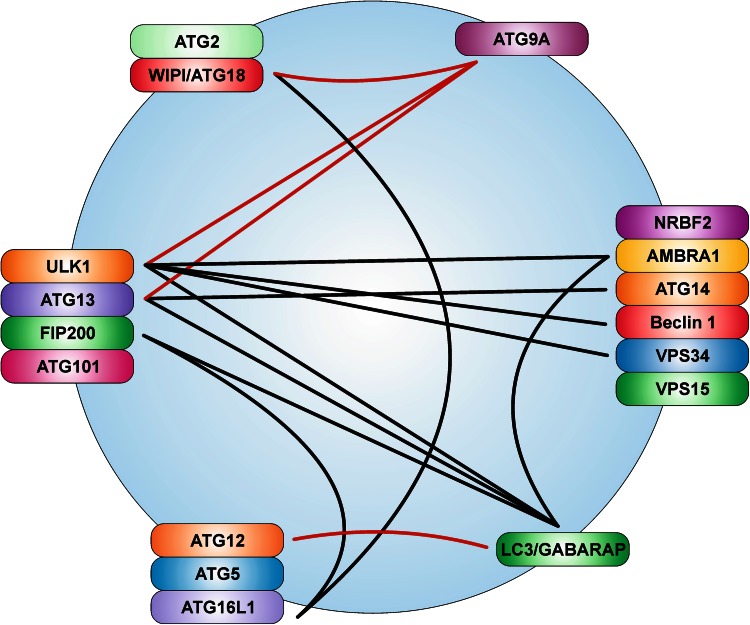
The ATG “spiderweb”. This scheme depicts the crosstalk between the six ATG signaling modules described in this review. The adjacent positioning of proteins within the single modules does not necessarily reflect direct interactions of the components. *Lines* can indicate both interaction and/or phosphorylation (by ULK1). Crosstalks identified for yeast orthologs are indicated by *red lines*

It appears that there is a direct crosstalk between the autophagy-initiating ULK1 protein kinase and VPS34/Beclin 1 lipid kinase complexes (Figs. 2, 3, 4). Russell et al. demonstrated that ULK1 directly phosphorylates Beclin 1 at S15 and thereby enhances the activity of the ATG14-containing VPS34 complexes (Fig. 3) [246]. This is further supported by the observation that ULK1 also phosphorylates AMBRA1 (Fig. 3) [162]. This phosphorylation triggers the dissociation of AMBRA1 and the associated PtdIns3K class III complex from dynein light chains 1/2. The resulting relocalization of this complex to the ER allows for the nucleation of autophagosomes [162]. Considering the results by Nazio et al. described in “The Atg1/ULK1 complex”, there apparently exists a mutual regulatory circuit involving ULK1 and AMBRA1, i.e., AMBRA1 regulates the stability and kinase activity of ULK1 by controlling its ubiquitination, and in turn ULK1 regulates the association of AMBRA1 with the cytoskeleton via phosphorylation. Recently, Egan et al. reported the direct phosphorylation of VPS34 by ULK1 [124].

There is not only crosstalk between the ULK1 complex and the VPS34/Beclin 1 complex, but these two autophagy-initiating complexes also share common upstream regulators, such as AMPK, mTOR and AKT (Fig. 3). AMPK phosphorylates both Beclin 1 and VPS34, respectively [247]. Apparently, AMPK inhibits the non-autophagy VPS34 complex by phosphorylating T163/S165 in VPS34, but activates the pro-autophagy VPS34 complex by phosphorylating Beclin 1 at S91/S94 [247]. Additionally, mTORC1 inhibits the PtdIns3K activity of the ATG14-containing VPS34 complex by phosphorylating ATG14 [248]. Finally, AKT directly phosphorylates Beclin 1 and inhibits autophagy by the formation of a phospho-Beclin 1/14-3-3/vimentin intermediate filament complex [249], and active EGFR binds Beclin 1, leading to its multisite tyrosine phosphorylation [196]. This phosphorylation decreases VPS34 catalytic activity, thereby establishing a direct link between oncogenic receptor tyrosine kinases and the autophagy machinery.

The product of the PtdIns3K class III catalytic activity is phosphatidylinositol 3-phosphate (PtdIns3P). This lipid then recruits further downstream effectors such as DFCP1 (see “Types of autophagy and morphology”) and proteins of the Atg18/WIPI-family (see “Atg18/WIPI proteins and Atg2/ATG2”) (Fig. 3). This has been confirmed by two studies, showing that knockdown of ATG14 or VPS34 leads to the disappearance of DFCP1- or WIPI1-positive puncta, respectively [250, 251]. However, it has been observed that autophagy might also be induced independently of VPS34/Beclin 1 [252, 253]. On the one hand this might be explained by alternative cellular sources of PtdIns3P, e.g., through class II PtdIns3Ks [254] or class I PtdIns3Ks in combination with the lipid phosphatases SHIP and INPP4 [255]. On the other hand, Rubinsztein and coworkers recently demonstrated that PtdIns5P can regulate autophagy via PtdIns3P effectors [256].

### Atg9/ATG9A

Atg9 is the only multi-spanning transmembrane protein among the Atgs (reviewed in [257, 258]) (Fig. 1, panel 3). In yeast, it was demonstrated that Atg9 concentrates in clusters that comprised vesicles and tubules, and that these compartments contribute to the de novo formation of the PAS [259]. Yamamoto et al. reported that single-membrane and Golgi-derived Atg9-vesicles with a diameter of 30–60 nm (containing approximately 30 Atg9 molecules) assemble to the PAS upon starvation [260]. These vesicles apparently become part of the phagophore and the outer autophagosomal membrane. Upon autophagosome completion, Atg9 clusters are recycled back to the cytoplasm [260]. It has been shown earlier that Atg9 is recruited to the PAS by Atg17 and that Atg9 cycling depends on Atg1–Atg13 and Atg18–Atg2 complexes, respectively [67, 261]. Additionally, several groups identified proteins that are involved in Atg9 trafficking, including Atg11, Atg17, Atg23, Atg27, Trs85, Arp2/3 and actin [262]. Recently, Backues et al. identified a core minimal machinery necessary and sufficient for the trafficking of Atg9 to the PAS, i.e., Atg11, Atg19, Atg23 and Atg27 [262]. Finally, Suzuki et al. recently reported that the Atg13 HORMA domain (described in “The Atg1/ULK1 complex”) can interact with Atg9 and thus recruits Atg9 vesicles during autophagosome formation (Fig. 4) [72]. As described above, Atg1-dependent phosphorylation of Atg9 is apparently important for the efficient recruitment of Atg8 and Atg18 to the site of autophagosome formation (Fig. 4) [68]. However, it has also been noted that the Atg9-positive vesicles described above are unlikely a major supplier of lipids for autophagosome biogenesis and thus likely possess an additional important function for autophagosome biogenesis [260].

In 2006, it has been reported that the mammalian Atg9 ortholog ATG9A (also referred to as mAtg9 or Atg9L1) is localized in the trans-Golgi network and in early, late and recycling endosomes [263, 264]. Upon starvation, ATG9A is redistributed to peripheral, endosomal membranes positive for the autophagosomal marker GFP-LC3 (see “Two ubi-quitin-like conjugation systems in autophagy: Atg12/ATG12–Atg5/ATG5 and Atg8/LC3–PE”) [264]. Like in yeast, this redistribution depends on the mammalian Atg1 homolog ULK1 [264]. An ATG9A compartment in mammals has been proposed which is similar to the one observed in yeast [263]. The authors suggest that ATG9A resides on a distinct tubular-vesicular compartment, and that this “ATG9A reservoir” continuously emanates from vacuolar recycling endosome-like structures by tubulation. They observed that subcellular ATG9A localization is regulated by ULK1 and WIPI2. However, ULK1 and WIPI2 are not required for the recruitment of ATG9A to early DFCP1-positive omegasomes [263]. Similar observations were previously made by other groups. For example, it has been demonstrated that ATG9A and ULK1 independently localize to the autophagosome formation site during canonical autophagy and Parkin-mediated mitophagy (see “Two ubiquitin-like conjugation systems in autophagy: Atg12/ATG12–Atg5/ATG5 and Atg8/LC3–PE”), and that ATG9A and ULK1 are independently recruited to *Salmonella*-containing vacuoles during xenophagy [265, 266]. Although ATG9A is essential for the formation of phagophores, it appears that ATG9A only transiently interacts with autophagosomes and does not integrate into the autophagosomal membrane [263]. Recently, Popovic and Dikic reported that TBC1D5 and the AP2 complex are important novel regulators of ATG9A trafficking towards the sites of autophagosome formation [267]. Finally, Puri et al. reported that ATG9A localizes on the plasma membrane in clathrin-coated structures and is internalized through early and then recycling endosomes [268]. Notably, the authors describe that ATG16L1 (see “Two ubiquitin-like conjugation systems in autophagy: Atg12/ATG12–Atg5/ATG5 and Atg8/LC3–PE”) is also internalized by clathrin-mediated endocytosis but via different clathrin-coated pits. The ATG9A- and ATG16L1-containing vesicles then “meet” in recycling endosomes in a VAMP3-dependent manner [268].

### Atg18/WIPI proteins and Atg2/ATG2

The Atg18 proteins constitute the second important family of PtdIns3P effectors (Fig. 1, panel 4). Whereas in yeast three family members have been identified so far, i.e., Atg18, Atg21 and HSV2/Ygr223c, in mammals four Atg18 homologs have been isolated, i.e., WD-repeat protein interacting with phosphoinositides 1–4 (WIPI1-4) [8, 269, 270]. DFCP1 binds to PtdIns3P via its FYVE domain, which was named after the first four proteins shown to contain it, i.e., Fab1, YOTB/ZK632.12, Vac1, and EEA1 [271]. In contrast, the Atg18/WIPI proteins bind to PtdIns-3P (and PtdIns(3,5)P_2_) via a seven-bladed β-propeller. Accordingly, the three yeast proteins and the four WIPIs have been called “PROPPINs” [272]. These proteins are WD40-repeat containing proteins and require an FRRG-motif for PtdIns3P-binding [270, 273, 274]. Two groups reported the crystal structure of yeast HSV2/Ygr223c, and these works indicate that there are two phosphoinositide binding sites in PROPPINS [275, 276]. Yeast Atg18 is important for autophagy, whereas Atg21 and HSV2/Ygr223c are rather involved in the Cvt pathway and in micronucleophagy, respectively [8, 277–279]. In 2010, Nair et al. reported that Atg18 and Atg21 facilitate the recruitment of Atg8–PE to the site of autophagosome formation [280]. During autophagy, Atg18 is in complex with Atg2 [281], and it was demonstrated that autophagosome formation can be achieved in the absence of Atg18 by expressing engineered PAS-targeted Atg2 [282]. In mammals, WIPI1 and WIPI2 share highest homology to Atg18 and have thus been reported to be involved in autophagy [283]. During autophagosome biogenesis, WIPI1/2 colocalize with the Beclin 1/VPS34 complex component ATG14, but not with the second PtdIns3P effector DFCP1 [8, 250]. It has been suggested that WIPI1/2 localize to the phagophore, whereas DFCP1 localizes to the omegasome [8]. Subsequently, it was demonstrated that WIPI2 positively regulates LC3-lipidation (see “Two ubiquitin-like conjugation systems in autophagy: Atg12/ATG12–Atg5/ATG5 and Atg8/LC3–PE”) and thus obviously contributes to the maturation process of omegasomes to autophagosomes [283]. Recently, Dooley et al. demonstrated that ATG16L1 directly binds WIPI2b (one of five WIPI2 isoforms) (Fig. 4) [284]. They observe that WIPI2b is a PtdIns3P effector upstream of ATG16L1 and is required for LC3 conjugation [284].

Two mammalian Atg2 homologs have been identified, ATG2A and ATG2B, and their simultaneous silencing causes a block in the autophagic flux [285]. Interestingly, ATG2A/B also regulate lipid droplet morphology [285].

### Two ubiquitin-like conjugation systems in autophagy: Atg12/ATG12–Atg5/ATG5 and Atg8/LC3–PE

#### Components of the two ubiquitin-like conjugation systems

Two ubiquitin-like conjugation systems are centrally involved in the expansion of autophagosomes: (1) Atg12/ATG12–Atg5/ATG5 system and (2) Atg8/LC3-phosphatidylethanolamine (PE) system (reviewed in [286]) (Fig. 1, panels 5 and 6). Within these conjugation systems, Atg12 and Atg8 represent the ubiquitin-like proteins, which are conjugated by E1-, E2- and E3-like enzymatic activities to Atg5 and PE, respectively. Within the first system, Atg12 is activated by the E1-like enzyme Atg7. Subsequently Atg12 is transferred to the E2-like Atg10 and irreversibly conjugated to K149 (yeast sequence; corresponds to human K130) of Atg5 [286–289]. The Atg12–Atg5 conjugate interacts with Atg16, and this complex forms a homo-oligomer [286, 290, 291]. All yeast Atgs involved in the Atg12–Atg5 conjugation system have mammalian counterparts with identical or similar functions, including an Atg16-like protein (ATG16L1) [286, 292–295]. In yeast, this multimeric complex has a molecular weight of approximately 350 kDa, and it has been suggested that the complex consists of an Atg12–Atg5–Atg16 tetramer [290]. In mammals, the complex eluted in fractions corresponding to 400 and 800 kDa, indicating that it might be composed of four or eight sets of ATG12–ATG5–ATG16L1 [292]. However, a crystallographic study combined with analytical ultracentrifugation experiments revealed that yeast Atg16 forms a parallel coiled-coil dimer [296]. The E1-like ATG7, the E2-like ATG3 and the conjugation acceptor ATG5 are essential for autophagy, and neonates of *Atg7*^−/−^, *Atg3*^−/−^ and *Atg5*^−/−^ mice die at day 1 after birth due to the neonatal starvation period [297–299]. Within the second conjugation system, Atg8 is activated by the common E1-like enzyme Atg7 and then transferred to the E2-like Atg3 [286, 300]. However, prior to Atg8 activation by Atg7, the C-terminal R117 has to be removed by the proteolytic activity of Atg4 in order to expose G116 [286, 301]. Interestingly, it has been demonstrated that the Atg12–Atg5 conjugate possesses an E3-like activity for Atg8 conjugation to PE [302, 303]. Although Atg16 is not important for efficient Atg8–PE conjugation in vitro, it is required for Atg8–PE formation in vivo [303]. Atg16 recruits Atg12–Atg5 to the PAS and thus determines the site of Atg8 lipidation [15, 51]. Two structural reports further support this link between the two conjugation systems. First, the crystal structure of Atg12–Atg5 indicates that Atg12 serves as binding module for the E2-like Atg3, essentially facilitating the transfer of Atg8 from Atg3 to the PE in the membrane [304]. Second, apparently the Atg12–Atg5 conjugate enhances the E2 activity of Atg3 by rearranging its catalytic site [305].

In the mammalian system, so far nine Atg8 orthologs have been reported. These can be subdivided into two families: (1) the LC3 subfamily consisting of microtubule-associated proteins 1A/1B light chain 3A (MAP1LC3A or briefly LC3A; two splice variants), LC3B, LC3B2, and LC3C, and (2) the GABARAP/GATE16 subfamily consisting of the γ-aminobutyric acid receptor-associated protein (GABARAP), GABARAPL1 (also termed ATG8L or GEC1), Golgi-associated ATPase enhancer of 16 kDa (GATE16, also termed GABARAPL2) and GABARAPL3 [306–315]. Additionally, four different mammalian ATG4 isoforms have been identified, i.e., ATG4A-D (also referred to as autophagin-1–4) [316, 317]. LC3B is probably the most extensively studied mammalian ATG8 protein, and it is cleaved C-terminally of G120 within the first 6 min of synthesis [309, 318]. ATG4-mediated cleavage of LC3 generates a cytosolic truncated LC3-I fragment of 18 kDa, which lacks the 22 C-terminal amino acids of the pro-form [318]. Interestingly, the different ATG4 isoforms possess selective preferences regarding their ATG8 family substrates [309, 319]. Subsequently, LC3-I is converted to the lipidated LC3-II isoform in an E1/E2/E3-cascade similar to the yeast system [295, 309, 320]. Accordingly, the mammalian ATG12–ATG5 conjugate interacts with ATG16L1, which targets the conjugate to the phagophore [292, 321]. There the ATG12–ATG5–ATG16L1 complex exerts its E3-like function and thus determines the site of LC3 lipidation [322]. This has been supported by the observation that forced expression of ATG16L1 at the plasma membrane led to ectopic LC3 lipidation at that site [322]. Similarly to LC3 conversion, the other mammalian ATG8 family members are processed by ATG4 isoforms, form E1- and E2-intermediates with ATG7 and ATG3, and are targeted to the autophagosome [295, 309, 312, 320, 323].

Next to cleavage and lipidation, it has also been reported that LC3 becomes phosphorylated. Jiang et al. observed that T9 and T29 of LC3 can be phosphorylated by PKC [324]. However, mutations of these residues to either alanine or aspartate/glutamate did not affect autophagy. In contrast, Cherra et al. reported the PKA-mediated phosphorylation of LC3 at S12, and this phosphorylation regulates the incorporation of LC3 into the autophagosomal membrane: the pseudophosphorylated S12D mutant showed reduced recruitment to autophagosomes, whereas the nonphosphorylatable S12A mutant exhibited enhanced puncta formation [325]. Of note, Atg8 orthologs of yeast and *D. melanogaster* lack this PKA site, and it is also absent in the mammalian GABARAP/GATE16 subfamily [325].

#### Selective autophagy and autophagy receptors/adaptors

Both conjugates, i.e., ATG12–ATG5 and Atg8/LC3–PE, are targeted to membranes during the autophagic process. Whereas ATG12–ATG5–ATG16L1 is mainly found at the phagophore [292, 321], Atg8/LC3–PE is present on the autophagosomal membrane throughout the whole process of vesicle biogenesis. The exact function of this “decoration” of the autophagosomal membrane is still intensely being investigated. In 2007, Nakatogawa et al. reported that Atg8 mediates the tethering and hemifusion of liposomes in vitro, and the authors suggested that this function contributes to the expansion of the phagophore in vivo [326]. Additionally, it has been reported that the amount of Atg8 determines the size of autophagosomes [327]. Generally it is tempting to speculate that the different Atg8 family proteins are selectively incorporated into the autophagosomal membrane depending on the autophagic stimulus, the step during the autophagic flux, or the cargo to be degraded. The latter two aspects are supported by two works. The first study proposes that LC3 proteins are rather involved in the elongation of the autophagosomal membrane, whereas GATE-16/GABARAP proteins function during the later stages of autophagosome maturation [314]. The second work reports that LC3C is required for efficient xenophagic clearance of *Salmonella typhimurium* [328]. This observation leads over to the best studied function of ATG8 family proteins, i.e., enabling the cell to differentially handle the cargo during selective autophagy. In recent years, a new class of cargo-recognition receptors has been identified and characterized, and they have been termed autophagy receptors [329–334]. These autophagy receptors are centrally involved in the recognition of cargo during selective autophagy processes, e.g., mitophagy or xenophagy. In 2005, Bjørkøy et al. discovered that p62 (alternatively called sequestosome 1, SQSTM1) forms protein aggregates which are degraded by autophagy [335]. In turn, inhibition of autophagy resulted in an increase of p62 protein levels. The authors suggested that p62 links polyubiquitinated proteins to the autophagic machinery via LC3. The same group could demonstrate that p62 directly binds to Atg8/LC3 [336]. They found an evolutionarily conserved 22-residue amino acid sequence within p62 which mediates the binding to LC3. This region was dubbed the LC3-interacting region (LIR), LC3 recognition sequence (LRS), or Atg8-family interacting motif (AIM), respectively [336–338]. Johansen et al. compiled a sequence logo from 25 different LIR motifs from 21 different proteins. It appears that LIR motif contains eight amino acids and is X_−3_X_−2_X_−1_W_0_X_1_X_2_L_3_ [330]. In this sequence, W might be replaced by F or Y (aromatic residue), L by I or V (large, hydrophobic residue), and acidic amino acids are frequently found in the X_−3_X_−2_X_−1_ positions. This suggestion was later confirmed by a compilation of 26 published LIR sequences [339]. Next to the LIR, p62 possess an ubiquitin-associated (UBA) domain and a Phox and Bem1p (PB1) domain, through which p62 can homo-oligomerize or bind to protein kinases. Accordingly, Johansen et al. proposed three required features of autophagy receptors: (1) existence of a LIR motif, (2) specific recognition of cargo, and (3) ability to polymerize [330]. Interestingly, Itakura et al. demonstrated that the targeting of p62 to the autophagosome formation site depends on the ability to self-associate, but not on LC3 or any other classical ATG [340]. The authors suggest that subsequently p62 oligomers are incorporated into autophagosomes in an LC3-dependent manner. In addition to p62, several additional autophagy receptors have been identified to date, including neighbor of breast cancer 1 (NBR1), optineurin (OPTN), nuclear domain 10 protein 52 (NDP52; alternatively termed Ca^2+^-binding and coiled-coil domain-containing protein 2, CALCOCO2), Toll interacting protein (TOLLIP), or cellular Casitas B-lineage lymphoma (c-Cbl) [341–345] (reviewed in [329–334, 346]).

Next to autophagy receptors which interact with both ubiquitin and LC3, other proteins have been identified which contribute to selective autophagy processes. These include proteins which interact with ubiquitin (e.g., HDAC6), which bind to LC3 (e.g., NCOA4, BNIP3, Nix, FUNDC1, BCL2L13, or FAM134B), or which indirectly associate with ubiquitinated proteins or LC3 (e.g., Alfy, BAG3, or Tecpr1) (reviewed in [333, 346]). FAM134B is presumably the mammalian ortholog of yeast Atg40 and is involved in ER-phagy [347]. Nuclear receptor coactivator 4 (NCOA4) was shown to mediate the delivery of ferritin to lysosomes [348]. BNIP3, Nix, FUNDC1 and BCL2L13 are mitochondrial proteins and are involved in the removal of mitochondria from maturing reticulocytes, during hypoxia-induced mitophagy, or induce mitochondrial fragmentation and mitophagy. In contrast, mitophagy of damaged mitochondria involves the action of PTEN-induced putative protein kinase 1 (PINK1) and the E3 ubiquitin ligase Parkin (reviewed in [349–351]). It has been suggested that Parkin hyper-ubiquitinates targets in the outer mitochondrial membrane, which are then recognized by autophagy receptors. The Parkin-dependent ubiquitylome in response to mitochondrial depolarization has been reported, and the authors found depolarization-dependent association of Parkin with numerous targets of the mitochondrial outer membrane, autophagy receptors, and the proteasome [352]. Interestingly, the autophagy receptors p62, NDP52, and Tax1BP1 were found to be depolarization-dependently ubiquitinated and associated with Parkin [352]. However, the involvement of p62 in damage-induced mitophagy is controversially discussed (reviewed in [349–351]). It appears that p62 is recruited to ubiquitinated cargo at damaged mitochondria and contributes to mitochondrial clustering, but its exact contribution to the mitophagic process itself awaits further clarification. Next to p62, it has been reported that optineurin is an autophagy receptor for damaged mitochondria in parkin-mediated mitophagy [353]. During the last 2 years additional mechanistic details have been deciphered with regard to PINK1/Parkin-dependent mitophagy. Activated PINK1 phosphorylates both Parkin and ubiquitin at S65, and these phosphorylations relieve the autoinhibition of Parkin, leading to an active phospho-ubiquitin-dependent E3 ligase and a feedforward signaling amplification loop [354–356]. Recently, Lazarou et al. suggested that NDP52 and optineurin are directly recruited to damaged mitochondria by PINK1-generated phospho-ubiquitin and thus support both Parkin-dependent and -independent mitophagy [357]. Generally, the interplay between these different routes of mitophagy (i.e., BNIP3/NIX/FUNDC1/BCL2L13 and PINK1/Parkin) is not entirely clarified. Furthermore, there likely exist additional pathways like the cardiolipin-mediated removal of injured mitochondria in neurons or the recently reported AMBRA1-dependent mitophagy, which can be both Parkin-dependent and -independent but requires LC3-binding (Fig. 4) [358, 359]. So far, the complementarity or even redundancy of the different mitophagy mechanisms remains elusive.

Future studies will have to delineate how ubiquitin signals regulate the selection of autophagy cargo. It is likely that additional autophagy receptors will be identified in the future, and next to “classical” autophagy receptors other forms of receptors will emerge. For example, cargo recognition by Tecpr1 is ubiquitin-independent. Instead, Tecpr1 binds to ATG5 and WIPI2 [139, 360]. Additionally, post-translational modifications such as phosphorylation might influence the function of autophagy receptors and presumably the cargo selection process, as shown for p62 and optineurin [345, 361, 362]. Finally, recently it has been demonstrated that N-terminal arginylation of the ER chaperone BiP is induced by cytosolic misfolded proteins [363]. Furthermore, cytosolic arginylated BiP binds to both the misfolded proteins and the ZZ domain of p62, leading to p62 aggregation, increased p62-binding to LC3, and targeting to autophagosomes [363]. Next to autophagy receptors, the term autophagy adaptor has been established [346]. Autophagy adaptors are ATG8 family-binding proteins which serve as anchors for the autophagy signaling machinery in order to facilitate autophagosome initiation, elongation, transport and fusion to lysosomes [346]. Accordingly, autophagy adaptors include components of the ULK1-complex (see below) and the Beclin 1/VPS34-complex, proteins of the ubiquitin-like ATG5/ATG12 and Atg8/LC3 conjugation systems, and proteins of the autophagosome–lysosome fusion machinery [346]. Recently, Wollert and colleagues described that Atg8–PE directly recruits Atg12–Atg5 conjugates through a non-canonical AIM in Atg12 (Fig. 4) [364]. Then Atg16 initiates a membrane scaffold by crosslinking Atg8–PE/Atg12–Atg5 complexes into a 2D meshwork [364]. The authors postulate that cargo receptors compete with Atg12–Atg5 for Atg8–PE binding, thus explaining why Atg8–PE can function both as a membrane scaffold and as cargo receptor [364].

Apparently there is also crosstalk between the ULK1 complex and components of the ubiquitin-like conjugation systems (Figs. 2, 4). Three articles focusing on the interaction between Atg1/ULK1 and Atg8 family proteins have been published in the recent past. Notably, the association of ULK1 with Gate16 and GABARAP was already described in the year 2000 by Okazaki et al., and they mapped the interaction sites to ULK1 amino acids 287–416 [365]. In the first of the three mentioned publications, Kraft et al. showed that Atg8 binds to a LIR within Atg1 [57]. They suggest that Atg8 targets Atg1 to autophagosomes where it might contribute to autophagosome maturation and/or their fusion to lysosomes. Additionally, the Atg8–Atg1 interaction targets the Atg1 complex for vacuolar degradation. The authors also demonstrated that mammalian ULK1 harbors a LIR and that ULK1 interacts with GABARAP, Gate16 and LC3B, thus directly confirming the results previously obtained by Okazaki et al. Similar to their observations in yeast, ULK1 was LIR-dependently targeted to autophagosomes [57]. In the second manuscript, Nakatogawa et al. independently confirmed the association between yeast Atg8 and Atg1 [366]. Mutation of the Atg8 family interacting motif (AIM) of Atg1 abolishes Atg1 transport to and degradation in vacuoles. Interestingly, AIM mutation caused a significant defect in autophagy, but did not affect PAS organization or the initiation of phagophore formation [366]. This result suggests that there are indeed two functions of Atg1/ULK1: (1) initiation of phagophore formation and (2) autophagosome expansion/maturation and/or fusion to vacuoles/lysosomes. Finally, the ULK1 LIR domain (D^356^FVMV) was also described by Alemu et al. [339]. Notably, they additionally found LIR sequences within ATG13 (D^443^FVMI) and FIP200 (D^701^FETI). Apparently, all three components of the ULK1 complex have preferences towards the GABARAP-subfamily of mammalian ATG8 proteins. As described above, the GATE-16/GABARAP family was reported to play a role in later stages of autophagy, indirectly suggesting that the ULK1 complex components have a function at a later stage, too. Alemu et al. verified that the LIR motif in ULK1 is required for the association of ULK1 with phagophores and/or autophagosomes. However, in contrast to the two reports by Kraft et al. and Nakatogawa et al., they state that ULK1 is mainly degraded by the proteasome with only marginal contribution from autophagy, which is in accordance to observations made by Joo et al. [123]. Another level of crosstalk between the ULK1 kinase complex and the ubi-quitin-like conjugation systems was deciphered recently by three works. Gammoh et al. and Nishimura et al. reported that FIP200 can directly interact with ATG16L1 [94, 103]. Gammoh et al. mapped the binding site within ATG16L1 to amino acids 229–242 and named this region FIP200-binding domain (FBD). Importantly, deletion of the FBD does neither abolish ATG16L1-binding to ATG5 nor self-dimerization. Expression of the FBD-deleted ATG16L1 in the ATG16L1-negative background could not fully reconstitute autophagy induced by amino acid starvation or the mTOR inhibitor Torin1 [94]. Similar results were reported by Nishimura et al. [103]. They showed that the FIP200–ATG16L1 interaction is independent of ATG14 or PtdIns3P, and that the interaction is important for ATG16L1 targeting to the phagophore. The authors narrowed down the interaction domain to two regions of ATG16L1, i.e., amino acids 230–250 (roughly overlapping with the FBD reported by Gammoh et al.) and 288–300. They additionally suggest that the ATG12–ATG5–ATG16L1 complex and the ULK1–ATG13–FIP200–ATG101 complex form one large unit in the cytoplasm, which then targets the phagophore. Accordingly, the authors describe their observation that ULK1 and ATG5 are recruited to the same compartment with similar kinetics (see “Molecular hierarchy of Atg/ATG proteins”). Most surprisingly, they observed a blockade of the autophagic flux when they express an ATG16L1Δ (230–300) mutant in *Atg16l1*^−/−^ MEFs, but not when expressing the ATG16L1 (1–230) version, which also lacks the FIP200-interacting domain. They proposed a model involving a self-inhibitory role for the C-terminal WD-repeat domain of ATG16L1. If this domain is deleted, the N-terminal half of ATG16L1 uncoordinatedly targets membranes, including the autophagosome formation site [103]. Finally, Lim et al. recently reported that ULK1 phosphorylates S405 and S409 within the ubiquitin-association (UBA) domain of p62 [361]. The S409 phosphorylation increases the binding affinity of p62 to ubiquitin. Collectively, the observations described above support a role of the ULK1 complex not only for autophagy initiation, but also for later steps of the autophagic cascade.

#### ATG8 proteins and monitoring autophagy

The detection of PE-conjugated GFP-LC3 at the autophagosomal double-membrane by confocal laser scanning microscopy is an established method for the analysis of autophagic processes [367]. This method has even been optimized by using the tandem fluorescence mRFP-EGFP-LC3 fusion protein [368]. Upon fusion of mature autophagosomes with lysosomes, the resulting low pH quenches the GFP fluorescence and accordingly autolysosomes can be detected as RFP-only structures [368]. By this means, the autophagic flux can be monitored. An improved tandem fluorescence-tagged mTagRFP-mWasabi-LC3 has been described by Zhou et al. [369]. Alternatively, the conjugation of LC3 to PE can also be detected by immunoblot analysis, since the lipidated LC3-II exhibits a slightly increased mobility in SDS-PAGE compared to the unlipidated LC3-I. In contrast to the Atg12/ATG12–Atg5/ATG5 conjugate, Atg8/LC3 proteins can be deconjugated from PE by the activity of Atg4/ATG4 isoforms [301]. It appears that this deconjugation is important to maintain an appropriate supply of Atg8/LC3 at early stages of autophagy, and to facilitate the maturation into fusion-capable autophagosomes at later stages [370]. Although the lipidation of LC3 is the basis for several standard detection methods of autophagy, several caveats have to be considered. Apparently, LC3 lipidation can occur in an autophagy-independent manner (reviewed in [371]). LC3-II can be detected in cells in which certain ATGs are deleted, e.g., in *Fip200*^−/−^ MEFs [82], *Becn1*^−/−^ ES cells [134], *BECN1*^−/−^ DT40 (own unpublished observation), *Ulk1*^−/−^ MEFs [83, 110], *Ulk1/2*^−/−^ MEFs [84, 372], or in cells in which certain ATGs are severely reduced by RNAi, e.g., Beclin 1 [134, 373], ATG13 [107, 109, 110], ULK2 [110], ATG14 [178], or VPS34 [178]. A similar observation was made for the yeast system. Atg8–PE was detected in yeast strains deficient for Atg1, Atg2, Atg6, Atg9, Atg13, Atg14, Atg16, or Atg17, and slightly also in strains deficient for Atg5 or Atg12 [15]. These observations indicate that LC3 is lipidated under conditions in which the autophagic flux is inhibited. Vice versa, a recent report by Szalai et al. suggests that autophagy of cytoplasmic bulk cargo does not require LC3 [374].

Finally, in 2007 Sanjuan et al. described that particles that engage TLRs on macrophages while they are phagocytosed trigger LC3 recruitment to the phagosome [375]. This processed was termed LC3-associated phagocytosis (LAP). LAP requires ATG5 and ATG7 and is preceded by Beclin 1 recruitment and PtdIns3K kinase activity [375]. Importantly, LC3 recruitment to the phagosomes was not associated with observable double-membrane structures. Next to TLR ligand-coated particles, LAP was observed upon phagocytosis of beads with LPS, killed yeast, or *E. coli* bacteria [375]. This indicates that autophagy proteins contribute to the elimination of pathogens not only through canonical autophagy/xenophagy, but also through LAP. Finally, a similar decoration of single membrane structures with LC3 was also described for phagosomes containing apoptotic cells, macropinosomes, and entotic vacuoles [376, 377] (reviewed in [378]).

### Molecular hierarchy of Atg/ATG proteins

#### Genetic hierarchy of Atg/ATG proteins

The functional units established by the Atg/ATG proteins are recruited to the PAS or the phagophore in a defined order, and historically the analysis of the hierarchical appearance of Atgs/ATGs has been subject of several investigations. In 2007, Suzuki et al. reported the hierarchy of Atg proteins in PAS organization of yeast [8, 51]. Apparently, Atg17 functions as scaffold for the recruitment of the other Atgs to the PAS. Atg1–Atg13, Atg9 and the PtdIns3K complex I act in initial stages, whereas Atg18–Atg2, Atg16–Atg5–Atg12 and Atg8–PE units are recruited to the PAS subsequently. In 2013, Suzuki et al. fine-mapped the localization of Atgs during autophagosome formation in yeast [379]. The authors defined specific localization sites: (1) the vacuole-isolation membrane contact site (VICS), (2) the isolation membrane (IM), and (3) the edge of the IM close to the ER (IM edge). They showed that Atg13, Atg17 and the yeast PtdIns3K complex I localize to the VICS, whereas Atg1, Atg8 and the Atg16–Atg5–Atg12 complex label the VICS and the IM. Finally, Atg2–Atg18 and Atg9 localized at the IM edge [379].

Itakura et al. performed a hierarchical analysis of mammalian ATGs [250]. Upon starvation, ULK1, ATG14, WIPI1, LC3 and ATG16L1 are recruited to the identical compartment, whereas DFCP1 localizes adjacently to these ATGs. Apparently, the ULK1 complex is the most upstream unit, and this unit is required for the recruitment of the ATG14-containing PtdIns3K class III complex. Puncta formation of DFCP1 and WIPI1 requires the presence of FIP200 and ATG14. Finally, LC3 and the ATG16L1–ATG5–ATG12 complex are the most downstream units [250]. Later it could be shown that ATG9A and FIP200 independently localize to the autophagosome formation site, but that both are necessary for the recruitment of the downstream factors ATG14 and WIPI1 [265].

Notably, the ATG recruitment hierarchy appears to depend on the autophagic stimulus. For example, ULK1 and ATG9A localize independently to the autophagosome formation site during starvation-induced autophagy, mitophagy, and *Salmonella* xenophagy [265, 266]. In contrast, LC3 recruitment depends on FIP200 and PtdIns3K class III complex during starvation-induced autophagy, whereas LC3 recruitment to CCCP-depolarized mitochondria or *Salmonella*-containing vacuoles (SCVs) is independent of FIP200 or ATG9A, respectively [265, 266]. These data indicate that there exist differences between canonical autophagy and selective autophagy processes such as mitophagy or xenophagy. With regard to *Salmonella* xenophagy, Noda et al. proposed a “Three-axis model” for ATG recruitment [380]. In this model, ULK1 and ATG9A are recruited independently to SCVs. Within the third axis, LC3 is recruited to SCVs by the ATG16L1 complex, but this recruitment does not depend on the other factors. The authors propose that the SCVs might represent an alternative membrane target for ATG16L1 recruitment, which is not present during starvation-induced autophagy [380].

#### Temporal hierarchy of Atg/ATG proteins

The above described analyses of the genetic hierarchy of Atg/ATG proteins revealed the functional interdependence among the single components. However, this does not necessarily correspond to the temporal recruitment of Atg/ATG proteins to the autophagosome formation site. The temporal aspect of ATG recruitment—i.e., the timing of accumulation peaks among mammalian ATG proteins—was addressed by Koyama-Honda et al. [381]. This study indicates that ULK1 and ATG5 complexes were synchronously recruited, although they are differently positioned in the genetic hierarchy. This might be explained by the above-mentioned interaction between FIP200 and ATG16L1. Furthermore, this observation led the authors to reassess the dependency of ULK1/ATG5 recruitment on PtdIns3K activity. It appears that both ULK1 and ATG5 can be stabilized by PtdIns3P at early stages, and that the ULK1 complex but not the ATG5 complex can become partially PtdIns3P-independent at later stages of autophagy [381]. Finally, the authors observe that the different ATGs are recruited to pre-existing vacuole membrane protein 1 (VMP1)-positive structures of the ER, although it has previously been suggested that VMP1 functions at a later step of autophagy [250, 381].

#### Network of Atg/ATG proteins

In recent years, it became more and more evident that the genetic interdependence of different ATGs combined with the differential association and dissociation of ATGs from the phagophore/autophagosomal membrane allow for the plasticity of an ATG-dependent autophagic response. Accordingly, the molecular regulation of autophagy rather resembles a “spiderweb-like” network than a one-way signaling cascade (see Fig. 4). Along these lines, different “signaling and scaffolding platforms”, which orchestrate the interplay between Atg/ATGs and non-Atg/ATGs during autophagy, have been proposed. For example, the exocyst complex has been implicated as protein scaffold for the autophagy machinery. The exocyst is a hetero-octameric complex involved in post-Golgi trafficking and vesicle tethering to the plasma membrane [382]. Bodemann et al. discovered that two exocyst subcomplexes containing either Sec5 or Exo84 regulate starvation-induced auto-phagy [383]. In this model, the Sec5 exocyst subcomplex exhibits a perinuclear localization and harbors the ULK1 kinase and PtdIns3K class III complexes. However, this subcomplex is autophagy-inactive. Along these lines, mTORC1 associated with the Sec5-subcomplex. Upon nutrient-deprivation, the Ras-like small GTPase RalB is activated, interacts with the exocyst, and promotes the replacement of Sec5 by Exo84. The Exo84-subcomplex is autophagy-active, and serves as platform for catalytically active ULK1 and Beclin 1/VPS34 complexes. Furthermore, the autophagy-active Exo84-subcomplex is localized to cytosolic puncta, presumably representing sites of autophagosome formation [382, 383]. Whereas the exocyst represents a signaling platform during non-selective starvation-induced autophagy, there likely exist corresponding platforms during selective autophagy processes. Of note, Deretic and colleagues recently described two alternative platforms which are connected to xenophagic processes. They found that tripartite motif protein family (TRIM) members regulate selective autophagy in multiple ways [384, 385]. On the one hand, several TRIM family members can act as platforms to assemble ULK1- and VPS34/Beclin 1-complexes (“TRIMosomes”). On the other hand, TRIMs can also function as autophagy receptors which directly bind their cargo, as exemplified by TRIM5 [384, 385]. TRIM5 recognizes and targets HIV-1 for autophagic destruction by directly binding both the viral capsid and ATG8 family proteins [384]. By this means, TRIM5 combines scaffolding and receptor properties [384, 385]. Similarly, Deretic and colleagues reported that immunity-related GTPase family M protein (IRGM)—which is a risk factor in Crohn’s disease—can assemble the core auto-phagy machinery and link it to innate immunity receptors, collectively promoting antimicrobial autophagy [386]. In the future, additional scaffolding platforms are likely to be identified.

## Conclusion

Over the past 20 years, the signal transduction of auto-phagy has become a central research field in cell biology. The molecular understanding of this process has been initiated by the identification of Atg/ATG proteins, and at present the network character of this signaling machinery and the identification of scaffolding/signaling platforms are shifting into focus. Furthermore, the importance of selective vs non-selective bulk degradation of cytoplasmic cargo is more and more appreciated. Generally, autophagic responses are constituted by two different branches. The short-term regulation mainly relies on protein–protein interactions and post-translational modifications such as phosphorylation, ubiquitination, or acetylation. In contrast, the long-term regulation of autophagy depends on transcriptional alterations [387], and the crosstalk between these two regulatory systems represents another important field of research.

Autophagy and its dysregulation have been implicated in different human diseases or processes, such as cancer, neurodegeneration, microbial infections, or aging. It is entirely conceivable that the modulation of autophagic signaling pathways represents a therapeutic target for several of these (patho-)physiological settings. The detailed understanding of the autophagy signaling machinery and its crosstalk is of central importance for the design of targeted therapies. One important example is tumorigenesis. In recent years, the ambivalent role of autophagy for cancer development has been characterized. On the one hand, it was demonstrated that various ATGs suppress tumor growth and that accordingly different autophagy-compromised mice are tumor-prone, e.g., *Becn1*^*+/−*^ mice [388]. In addition to its tumor suppressing effects, it has been postulated that autophagy might function as an alternative cell death mechanism. Collectively, these data form the basis for various preclinical studies supporting autophagy induction for cancer treatment [389]. On the other hand, autophagy functions as a cyto-protective mechanism, and thus contributes to the survival of cancer cells under nutrient-deprived conditions frequently found in tumors or metastasizing cancer cells [390]. Additionally, it could be shown that these cyto-protective effects support the resistance of cancer cells to metabolic or genotoxic stress induced by hormonal deprivation, chemotherapy or radiation [6, 389]. Furthermore, some cancer types have been shown to be “autophagy addicted”, and they depend on autophagy even in the absence of external stresses (e.g., RAS-driven cancers) [391]. Accordingly, the disruption of autophagic signaling pathways has also evolved as a therapeutic strategy and is applied in many preclinical studies and ongoing clinical trials [389]. In summary, it has been proposed that the pro- and anti-tumorigenic potential of autophagy is tumor stage-dependent [392]. Taking this into consideration, therapies based on autophagy induction might be beneficial for the prevention of tumorigenesis or tumor progression, whereas treatments employing the inhibition of autophagy likely support tumor regression or enable the overcoming of therapy resistance [6].

So far, the availability of autophagy-inhibiting compounds used in preclinical studies or even clinical trials is very limited. Currently, all clinical trials pursuing the inhibition of autophagy make use of the antimalarial lysosomotropic drugs chloroquine or hydroxychloroquine, which inhibit the fusion of autophagosomes with lysosomes [389]. However, several non-autophagic effects of these compounds have been reported [389]. With regard to preclinical studies, additional autophagy-inhibitory regimens have been tested, including bafilomycin A_1_, ammonium chloride, 3-methyladenine, or siRNA against several essential ATGs [389, 391]. However, these compounds are not specific or cannot be employed for clinical trials. Recently, three different VPS34-selective inhibitors have been reported, i.e., VPS34-IN1, SAR405, and PIK-III [255, 393, 394]. For SAR405, the authors reported a synergistic antiproliferative activity in renal tumor cell lines in combination with the mTOR inhibitor everolimus [394]. With regard to the ULK1 complex, the structure of the ULK1 kinase domain in complex with multiple inhibitors has been reported [118]. Furthermore, recently the first ULK1/2 inhibitors have been reported and characterized, i.e., MRT67307/MRT68921 and SBI-0206965 [124, 395]. Similar to the results obtained with the VPS34-inhibitor SAR405, the ULK1-inhibitor SBI-0206965 synergizes with mTOR inhibition to induce cell death in A549 non-small cell lung cancer cells [124]. Generally, it has become evident that MTOR inhibitors and other anticancer drugs induce cytoprotective autophagy, ultimately leading to a compromised efficacy of these compounds. Therefore, combinatorial therapies employing these agents and parallel ULK1 and/or VPS34 inhibition represents a promising anticancer strategy. Collectively, the development of targeted therapies—based on the molecular understanding of the autophagy signaling machinery—will be a central task for the coming years.

## Electronic supplementary material

Supplementary material 1 (DOCX 26 kb)
