# Supplementary material for: Autophagy signal transduction by ATG proteins: from hierarchies to networks
Source: Cell Mol Life Sci. 2015 Sep 21;72(24):4721–57. doi: 10.1007/s00018-015-2034-8 (PMC4648967; doi:10.1007/s00018-015-2034-8)
Supplement: Supplementary file 1 — Supplementary material 1 (DOCX 26 kb) [file 18_2015_2034_MOESM1_ESM.docx]

**Supplemental Table 1: Published ULK1 phospho-sites**

**(human amino acid sequence, Uniprot ID** **O75385 )**

|  | Site | Dorsey  [[119](#_ENREF_119)] | Egan  [[140](#_ENREF_140)] | Kim  [[141](#_ENREF_141)] | Shang  [[121](#_ENREF_121)] | | Bach  [[128](#_ENREF_128)] | Mack  [[120](#_ENREF_120)] |
| --- | --- | --- | --- | --- | --- | --- | --- | --- |
| Kinase | S87 | X |  |  |  | |  |  |
|  | T180 |  |  |  |  | | x |  |
|  | S195 | X |  |  |  | |  |  |
|  | S224 | X |  |  |  | |  |  |
| P/S-rich domain | S317 |  |  | x |  | |  | x |
|  | S341 | x |  |  | X | |  |  |
|  | S403 |  |  |  |  | |  | x |
|  | S405 |  |  |  | X | |  |  |
|  | S411 |  |  |  |  | |  | x |
|  | S450 | X |  |  | X | |  | x |
|  | S467 | X | x |  |  | |  |  |
|  | T468 |  |  |  | X | |  |  |
|  | S469 | X |  |  | X | |  |  |
|  | S477 |  |  |  |  | |  | x |
|  | S479 | X |  |  | X | |  | x |
|  | *S482 | x |  |  |  | |  |  |
|  | S495 |  |  |  |  | |  | x |
|  | S505 |  |  |  | X | |  |  |
|  | S522 | x |  |  |  | |  | x |
|  | S533 |  |  |  | X | |  | x |
|  | S544 |  |  |  |  | |  | x |
|  | S556 |  | x |  | X | | x | x |
|  | T575 |  | x |  |  | |  |  |
|  | *S614 |  |  |  |  | |  | x |
|  | S623 | x |  |  | X | |  | X |
|  | T625 |  |  |  |  | |  | x |
|  | T636 |  |  |  |  | |  | x |
|  | S638 |  | x |  | X | X |  | x |
|  | T660 |  |  |  |  | |  | x |
|  | S694 |  |  |  |  | |  | x |
|  | T695 |  |  |  |  | |  | x |
|  | S696 |  |  |  |  | |  | x |
|  | S716 |  |  |  |  | |  | X |
|  | S719 |  |  |  |  | |  | X |
|  | S748 |  |  |  |  | |  | x |
|  | T755 |  |  |  |  | |  | x |
|  | S758 | x |  | x | x | |  | x |
|  | T764 |  |  |  |  | |  | x |
|  | S775 |  |  |  |  | | x | x |
|  | *S777 |  |  | x |  | |  | x |
|  | S781 |  |  |  |  | |  | x |
| CTD | S866 | X |  |  |  | |  |  |
|  | S912 | X |  |  |  | |  |  |
|  | S1042 | x |  |  |  | |  | x |
|  | T1046 | x |  |  |  | |  |  |
|  |  |  | |  |  | |  |  |
| Color code: | | AMPK | mTOR | ULK1 | AKT | | PKA |  |

(Residues indicated with * refer to the position in murine sequence, since the position is not conserved in human. P/S-rich domain, proline/serine-rich domain; CTD, C-terminal domain)
